# Adipose tissue hyaluronan production improves systemic glucose homeostasis and primes adipocytes for CL 316,243-stimulated lipolysis

**DOI:** 10.1038/s41467-021-25025-4

**Published:** 2021-08-10

**Authors:** Yi Zhu, Na Li, Mingyang Huang, Mason Bartels, Sophie Dogné, Shangang Zhao, Xi Chen, Clair Crewe, Leon Straub, Lavanya Vishvanath, Zhuzhen Zhang, Mengle Shao, Yongjie Yang, Christy M. Gliniak, Ruth Gordillo, Gordon I. Smith, William L. Holland, Rana K. Gupta, Bingning Dong, Nathalie Caron, Yong Xu, Yucel Akgul, Samuel Klein, Philipp E. Scherer

**Affiliations:** 1 Touchstone Diabetes Center, Department of Internal Medicine, The University of Texas Southwestern Medical Center at Dallas, Dallas, TX USA; 2 Children’s Nutrition Research Center, Department of Pediatric, Baylor College of Medicine, Houston, TX USA; 3 Department of Endocrinology and Metabolism, Tianjin Medical University General Hospital, Tianjin, China; 4 Department of Plastic Surgery, The University of Texas Southwestern Medical Center at Dallas, Dallas, TX USA; 5 Molecular Physiology Research Unit, NARILIS, University of Namur, Namur, Belgium; 6 Center for Human Nutrition, Washington University School of Medicine in St. Louis., St. Louis, MO USA; 7 Department of Nutrition and Integrative Physiology, University of Utah College of Health, Salt Lake City, UT USA; 8 Department of Molecular and Cellular Biology, Baylor College of Medicine, Houston, TX USA; 9 Department of Cell Biology, The University of Texas Southwestern Medical Center at Dallas, Dallas, TX USA

**Keywords:** Mechanisms of disease, Fat metabolism, Diabetes, Obesity

## Abstract

Plasma hyaluronan (HA) increases systemically in type 2 diabetes (T2D) and the HA synthesis inhibitor, 4-Methylumbelliferone, has been proposed to treat the disease. However, HA is also implicated in normal physiology. Therefore, we generated a Hyaluronan Synthase 2 transgenic mouse line, driven by a tet-response element promoter to understand the role of HA in systemic metabolism. To our surprise, adipocyte-specific overproduction of HA leads to smaller adipocytes and protects mice from high-fat-high-sucrose-diet-induced obesity and glucose intolerance. Adipocytes also have more free glycerol that can be released upon beta3 adrenergic stimulation. Improvements in glucose tolerance were not linked to increased plasma HA. Instead, an HA-driven systemic substrate redistribution and adipose tissue-liver crosstalk contributes to the systemic glucose improvements. In summary, we demonstrate an unexpected improvement in glucose metabolism as a consequence of HA overproduction in adipose tissue, which argues against the use of systemic HA synthesis inhibitors to treat obesity and T2D.

## Abstract (web summary)

Hyaluronan is a naturally occurring linear polysaccharide that together with collagens, enzymes, and glycoproteins forms the extracellular matrix. Here the authors show that adipose tissue overproduction of Hyaluronan reduces fat accumulation in mice fed high-fat diet and improves systemic glucose homeostasis.

## Introduction

The extracellular matrix (ECM) is a three-dimensional network composed of collagens, enzymes, and glycoproteins that provide structural and biophysical support for cells^1,2^. Non-sulfated glycosaminoglycans, such as hyaluronic acids (HA), are important components of the ECM as well. HA is a polysaccharide with simple linear repeating disaccharide units of d-glucuronic acid (GlcUA) and N-acetyl-d-glucosamine (GlcNAc)^3,4^, synthesized by hyaluronan synthases^5^ and broken down by hyaluronidases or via non-enzymatic degradation by reactive oxygen species^6^.

Glucose is the starting point of HA synthesis: cytosolic UDP-GlcUA and UDP-GlcNA, produced by side reactions of the glycolytic pathway, are used by hyaluronan synthases to produce HA. Accumulation of HA has been observed in both type 1 and type 2 diabetes and chronic liver diseases, either locally or systemically. Peri-islet HA deposition is associated with the loss of immune-tolerance and development of insulitis in type 1 diabetes (T1D)^7^. Increased plasma HA levels are also associated with type 2 diabetes and liver cirrhosis^8–10^. Thus, HA has been implicated as a causal factor for many metabolic diseases^11^.

On the other hand, HA plays important roles in many aspects of normal physiology. For example, it has a high abundance in dermal tissues and is crucial in retaining water content in the skin due to its extreme hydrophilicity^12^. HA is also crucial for joint health, acting as a natural lubricant for joints^13,14^. Recently, a HA constituted perineuronal net has been implicated in neuronal maturation^15^. HA injections have been approved for the treatment of knee pain caused by osteoarthritis in patients^16^. All these beneficial aspects of HA prompt many individuals to take HA as nutritional supplements or look for other ways to increase systemic HA levels.

Here, we started with the observations that metabolically unhealthy individuals with obesity and prediabetes have higher basal and postprandial circulating HA levels than people who are obese, but who are metabolically healthy and with normal oral glucose tolerance. To understand whether circulating HA levels directly drive metabolic dysfunction, we have used transgenic mouse models with altered HA levels in various tissues and in circulation. Contrary to our expectations, adipose tissue HA production significantly improved systemic glucose tolerance. We excluded the possibility that increased circulating HA that leaks from *Has2* overexpressing adipose tissue is responsible for the improvement in systemic glucose metabolism by using two other experimental models with elevated circulating HA. We determined that HA primes adipocytes for lipolysis, resulting in smaller adipocytes and overall lower body weight and lower fat mass upon a metabolic challenge. HA-driven lipolysis and decreases in lipid content are cell-autonomous phenomena, which can be replicated in vitro differentiated adipocytes, and in hepatocytes in vivo. Furthermore, assaying tissue HA levels raises some doubts regarding the effectiveness of 4-Methylumbelliferone (4-MU) to inhibit HA synthesis in vivo. Overall, we conclude that adipose tissue HA production exerts beneficial effects on adipose tissue and improves whole-body metabolic function.

## Results

### Circulating HA inversely correlates with metabolic health

**Fig. 1:**
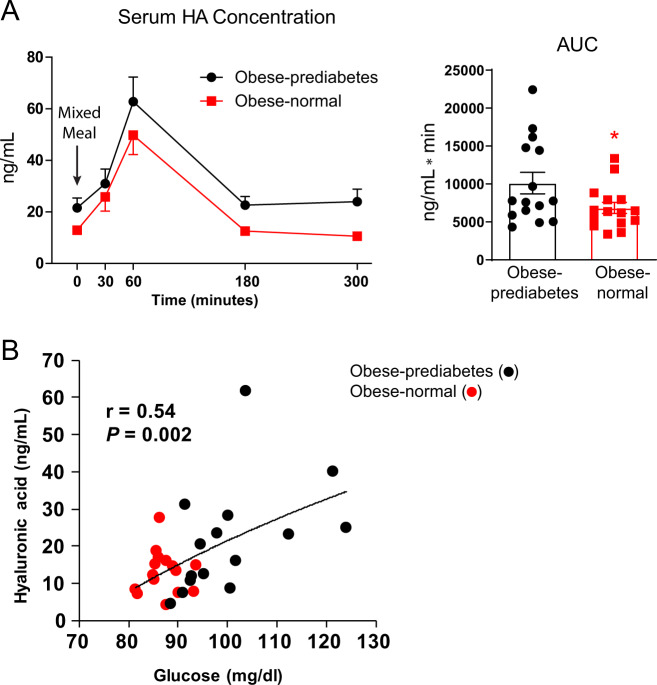
Circulating HA inversely correlates with metabolic fitness in the human. **A** Obese-prediabetes (Obese-prediabetes; *n* = 15 patients) patients have higher levels of circulating HA compared to obese-normal (Obese-normal; *n* = 15 patients) patients. Subjects ingested a mixed meal at the indicated time point. Mean ± s.e.m; For AUC, two-tailed *t*-test, *p* = 0.0499. *indicates *p* ≤ 0.05. **B** Plasma HA concentrations (*t* = 0’) were directly correlated with basal plasma glucose concentrations. Logarithmic regression analysis was used to determine the line of best fit to the data.

We evaluated plasma HA concentrations before and for 5 h after ingesting a standard meal (50% carbohydrate, 35% fat, 15% protein) containing one-third of their estimated energy requirements^17^ in people with obesity and prediabetes (“Obese-prediabetes”) and those with obesity and normal oral glucose tolerance (“Obese-normal)”, who were all matched for body mass index and percent body fat. Obese-prediabetic cohort has greater plasma glucose, insulin, HbA1C and triglyceride concentrations, an index of insulin resistance (homeostasis model of insulin resistance [HOMA-IR])^18^, and intrahepatic triglyceride content, and lower plasma HDL-cholesterol concentrations than in the Obese-normal group (Table 1). Both basal HA concentrations, postprandial plasma HA concentrations and plasma HA area under the curve (AUC) were greater in the Obese-prediabetic group than the Obese-normal group (Fig. 1A). In addition, basal plasma HA concentrations were directly correlated with basal plasma glucose concentrations (Fig. 1B). These data demonstrate an inverse relationship between plasma HA concentrations and metabolic health and implicate circulating HA as a possible causative factor for metabolic dysfunction. However, the observational design of this study cannot determine cause-and-effect relationships. HA levels fluctuate during the day and rapidly increase after a meal, possibly due to vasodilatation and increases in intestinal lymph flow that displace HA from gastrointestinal tissues^19,20^.

**Table 1:** Body composition and metabolic characteristics of study subjects.

	Obese-Nl (*n* = 15)	Obese-PD (*n* = 15)	*p*-value
Body mass index (kg/m^2^)	37.9 ± 1.2	39.1 ± 1.3	0.50
Body fat (%)	47.7 ± 1.4	47.7 ± 1.4	0.99
IHTG content (%)	1.7 ± 0.1	16.6 ± 2.5	<0.01
Glucose (mg/dL)	87 ± 1	101 ± 3	<0.01
Insulin (µU/mL)	13 ± 2	27 ± 3	<0.01
HOMA-IR	2.9 ± 0.4	7.0 ± 1.0	<0.01
HbA1C (%)	5.0 ± 0.1	5.7 ± 0.2	<0.01
Triglyceride (mg/dL)	78 ± 7	132 ± 18	<0.01
HDL-cholesterol (mg/dL)	55 ± 4	43 ± 3	0.01
LDL-cholesterol (mg/dL)	91 ± 6	108 ± 6	0.08

Data are expressed as mean ± SEM. Two-tailed *t*-test.

*HbA1C* hemoglobin A1C, *IHTG* intrahepatic triglyceride, *HOMA-IR* homeostasis model assessment of insulin resistance, *Obese-Nl* obese with normal oral glucose tolerance, *Obese-PD* obese with prediabetes.

### High-dose 4-methylumbelliferone (4-MU) treatment improves glucose tolerance independent of HA inhibition

**Fig. 2:**
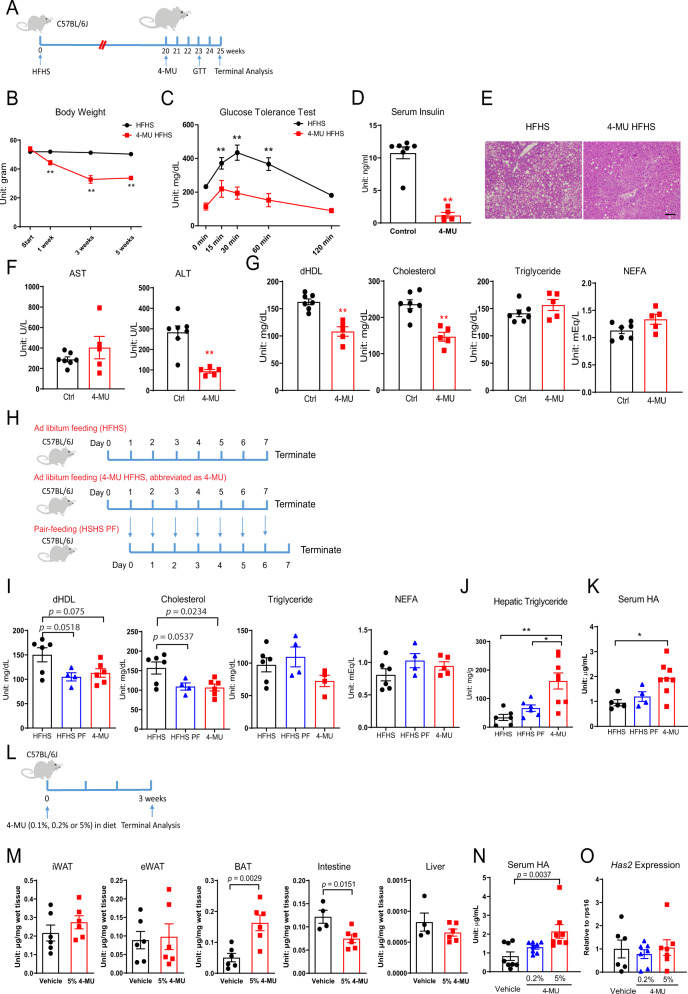
4-MU treatment on HA levels, glucose, and lipid metabolism. **A** Schematic representation of mouse treatment for panels **B**–**G**. **B** Body weight after 5 weeks of 4-MU HFHS treatment (*n* = 7 mice for HFHS, *n* = 6 mice for 4-MU HFHS treatment group). Two-way ANOVA followed by Sidak’s multiple comparisons test, adjusted *p*-value = 0.7473, 0.0006, <0.0001, <0.0001 for start, 1 week, 3 weeks and 5 weeks, respectively. **C** Glucose tolerance after 3 weeks of 4-MU HFHS treatment (*n* = 7 mice for HFHS, *n* = 6 mice for 4-MU HFHS treatment group). Two-way ANOVA followed by Sidak’s multiple comparisons test, adjusted *p*-value = 0.0646, 0.0084, <0.0001, 0.0001, 0.238 for 0, 15, 30, 60, and 120 min, respectively. **D** Fasting insulin levels after 5 weeks of 4-MU HFHS treatment (*n* = 7 mice for HFHS, *n* = 6 mice for 4-MU HFHS treatment group). Two-tailed *t*-test, *p* < 0.0001. **E** Representative pictures of hepatic lipid accumulation. Scale bar = 100 $\upmu$m. **F** Serum AST and ALT enzymatic activity after 5 weeks of 4-MU treatment (*n* = 7 mice for HFHS, *n* = 5 mice for 4-MU HFHS treatment group). Two-tailed *t*-test, *p* = 0.2547 and 0.0006 for AST and ALT, respectively. **G** Serum dHDL, Cholesterol, Triglyceride and NEFA levels after 5 weeks of 4-MU HFHS treatment (*n* = 7 mice for HFHS, *n* = 5 mice for 4-MU HFHS treatment group). Two-tailed *t*-test, *p* = 0.0002, 0.0005, 0.2057, 0.0691 for dHDL, Cholesterol, Triglyceride and NEFA, respectively. **H** Schematic representation of mouse treatment for panels **I**–**K**. The average amount of food eaten by mice on day “*N*” in ad libitum 4-MU HFHS feeding group will be given to pair-fed group on day “*N* + 1”. **I** Serum dHDL, Cholesterol, Triglycerides and NEFA levels (*n* = 6 mice for HFHS, *n* = 4 mice for HFHS PF, *n* = 8 mice for 4-MU treatment group). One-way ANOVA followed by Tukey’s multiple comparisons test. *p*-values are reported on the graph. **J** Hepatic triglyceride content (*n* = 6 mice for HFHS, *n* = 6 mice for HFHS PF, *n* = 8 mice for 4-MU treatment group). One-way ANOVA followed by Tukey’s multiple comparisons test. Adjusted *p* = 0.0014 for HFHS vs. 4-MU, adjusted *p* = 0.0139 for HFHS PF vs. 4-MU. **K** Serum HA levels (*n* = 5 mice for HFHS, *n* = 4 mice for HFHS PF, *n* = 8 mice for 4-MU treatment group). One-way ANOVA followed by Tukey’s multiple comparisons test. Adjusted *p* = 0.0131 for HFHS vs. 4-MU, adjusted *p* = 0.0851 for HFHS PF vs. 4-MU. **L** Schematic representation of mouse treatment for panels **M**–**O**. Multiple cohorts of mice were used. **M** Tissue HA levels from mice treated with 5% 4-MU in diet. iWAT: inguinal adipose tissue, eWAT: epididymal adipose tissue, BAT: brown adipose tissue (*n* = 6 mice for each group). Intestines and livers were harvested from a different cohort of mice undergoing the same treatment (*n* = 4 mice for vehicle treatment, *n* = 6 mice for 5% 4-MU treatment). Two-tailed *t*-test. **N** Serum HA levels from mice treated with 0.2% or 5% 4-MU HFHS (*n* = 8 mice for each group). One-way ANOVA followed by Tukey’s multiple comparisons test. **O** Adipose tissue *Has2* expression from mice treated with 0.2% or 5% 4-MU HFHS (*n* = 6 mice for control group, *n* = 7 mice for 0.2% 4-MU and 5% 4-MU). One-way ANOVA followed by Tukey’s multiple comparisons test. All data are presented as mean ± s.e.m. *indicates *p* ≤ 0.05, **indicates *p* ≤ 0.01.

To test whether elevated HA levels cause impaired metabolic homeostasis, 4-MU was used to inhibit HA production (Fig. 2A)^21–23^. Treatment of a cohort of obese mice fed with a high-fat/high-sucrose (HFHS) diet for 20 weeks, with subsequent exposure to 5 weeks of HFHS diet containing 4-MU, significantly reduced body weight (Fig. 2B). The treatment also significantly improved glucose tolerance (Fig. 2C) and reduced fasting insulin levels by more than 90% (Fig. 2D). Hepatic triglyceride accumulation was reduced after the treatment (Fig. 2E). Serum alanine transaminase activity was reduced (Fig. 2F), suggesting a potent protective effect of 4-MU treatment on the liver. Serum lipid profiles were also vastly improved: direct high-density lipoprotein (dHDL) and total cholesterol levels were significantly reduced (Fig. 2G).

A drawback of 4-MU treatment is that it has to be used at very high doses (5% by weight supplemented in diet)^24,25^ and it suppresses food intake at the beginning of the treatment (Supplementary Fig. 1A)^25^. Therefore, a pair-fed group was introduced in the second 4-MU treatment experiment ((Fig. 2H). After 1 week of 4-MU treatment, there was an average of 15.3% weight loss in 4-MU treated mice, and pair feeding led to a similar degree of weight loss (Supplementary Fig. 1B). Similar to the 5-week treatment, 1-week 4-MU exposure reduced serum dHDL and cholesterol, and a similar level of improvement was observed in the pair-fed group (Fig. 2I). Triglycerides and NEFA were not drastically different (Fig. 2I). 4-MU significantly increased hepatic triglyceride content compared to HFHS controls (Fig. 2J), possibly an effect of fasting resulting from reduced food intake. Surprisingly, 4-MU significantly increased circulating HA when compared to HFHS controls (Fig. 2K).

Upon reducing the content of 4-MU in HFHS diet to 0.1% (1 gram 4-MU per kg of diet), during a 3-week treatment regimen (Fig. 2L), mice consumed the same amount of 4-MU diet (Supplementary Fig. 1C), did not lose weight (Supplementary Fig. 1D) and there was no improvement in glucose tolerance (Supplementary Fig. 1E).

Surprisingly, 5% 4-MU also failed to reduce HA content in two different white adipose tissue depots, and increased HA content in the brown adipose tissue (Fig. 2M). Intestinal HA was significantly reduced by 5% 4-MU treatment (Fig. 2M). Liver had much lower (less than 1% compared to intestine) tissue HA, and the level was not affected by 4-MU treatment (Fig. 2M). As seen in pair-fed experiment, 5% 4-MU significantly increased serum HA levels (Fig. 2N) and had no effect on *Has2* expression in the inguinal adipose tissue (Fig. 2O).

### *Has2* overexpression increases high-molecular-weight (HMW) HA

Owing to the complex nature of the high-dose 4-MU treatment, and the surprising observation that it failed to reduce adipose tissue HA levels, but rather lead to an unexpected increase in circulating HA levels, we decided to develop genetic models to understand the role of HA in systemic metabolism in vivo.

**Fig. 3:**
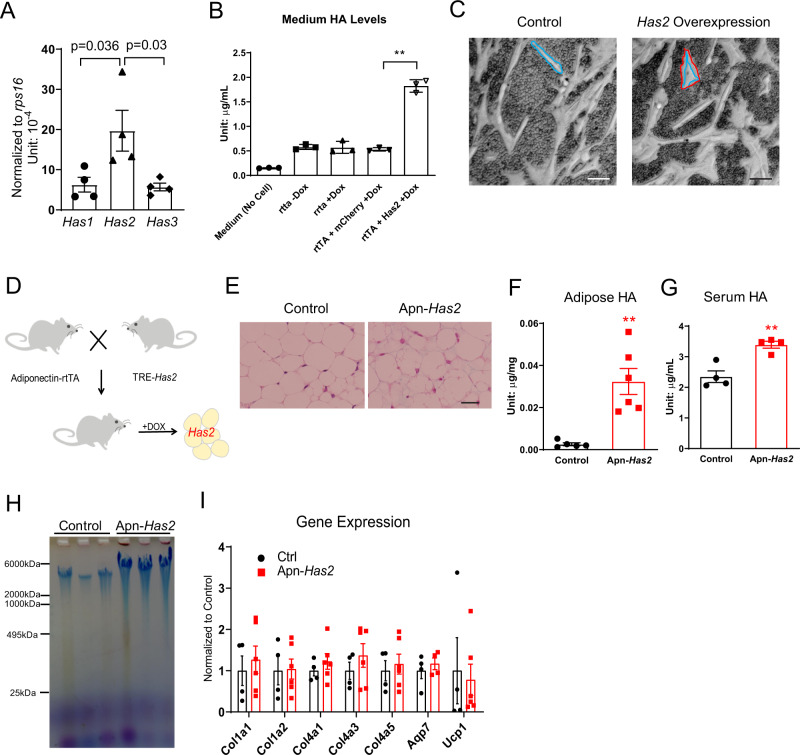
*Has2* overexpression increases HMW HA. **A***Has1*, *Has2*, and *Has3* expression levels in the adipose tissue from 16-week-old C57BL/6J mice fed with normal chow diet (*n* = 4). One-way ANOVA followed by Tukey’s multiple comparisons test. **B** HEK293 cells overexpressing *Has2* increase medium HA levels (*n* = 3). Two-tailed *t*-test to compare rtTA + mCherry + Dox vs. rtTA + Has2 + Dox, *p* < 0.0001. **C** Representative picture showing Met1 cells overexpressing *Has2* have increased extracellular space revealed by adding mouse red blood cells into the culture before the microscopic imaging. Blue line indicates the cell’s body, red line indicates the boundary of cell’s extracellular matrix. The space between two lines is the extracellular matrix. Scale bar = 50 µm. **D** Schematic of mouse cross to generate adipose tissue-specific doxycycline-inducible *Has2* overexpressing mice (Apn-*Has2*). **E** Representative pictures of inguinal adipose tissue slides stained with Alcian blue (Scale Bar = 50 µm). Mice were treated with Dox600 chow diet for 5 days. **F** Tissue HA concentration after adipose tissue *Has2* overexpression induced by Dox600 chow diet treatment for 5 days (*n* = 5 for control, *n* = 6 for Apn-*Has2* transgenic mice). Two-tailed *t*-test, *p* = 0.0019. **G** Serum HA concentration after adipose tissue *Has2* overexpression induced by Dox600 chow diet treatment for 5 days (*n* = 4). Two-tailed *t*-test, *p* = 0.0032. **H**  Electrophoresis of HA extracted from control and Apn-*Has2* mice treated by Dox600 chow diet for 5 days, demonstrating *Has2* overexpression mainly increases very high molecular weight HA. **I** Gene expression in adipose tissue overexpressing *Has2* after 5 days of Dox600 chow diet treatment (*n* = 4 for control, *n* = 6 for Apn-*Has2* mice). Two samples were not detected for *Aqp7* gene. Two-tailed *t*-test for each gene. All data are presented as mean ± s.e.m. **indicates *p* ≤ 0.01.

Based on the endogenous expression pattern, we chose to overexpress the most abundant adipose tissue hyaluronic acid synthase (*Has)* isoform, *Has2*, to increase HA levels (Fig. 3A and Supplementary Fig. 2A). The mouse cDNA encoding the ORF for the *Has2* gene was subcloned under the control of a tetracycline response element (TRE) promoter to make a TRE-*Has2* construct. In HEK293 cells with stable rtTA overexpression, *Has2* overexpression increased HA concentrations in the supernatant by more than threefold with 0.5 µg/mL doxycycline in the medium (Fig. 3B), reflecting that overexpression of a single HA synthase in mammalian cells is sufficient to promote HA production. Met1 mammary tumor cells stably transfected with CMV-rtTA and TRE-*Has2* construct also displayed increased extracellular space after 24-h 0.5 µg/mL doxycycline treatment (Fig. 3C). We generated TRE-*Has2* transgenic mice by injecting the linearized TRE-*Has2* construct into the pronuclear region of fertilized oocytes. Founders were crossed to adiponectin-rtTA (Apn-rtTA) mice^26^ for screening (Fig. 3D). A line that displays robust doxycycline-induced transgene expression with minimal levels of transgene leakage was chosen for further analysis (Supplementary Fig. 2B). Transgenic overexpression leads to a significant increase in adipose tissue HAS2 protein (Supplementary Fig. 2C). After 5 days of transgene induction on 600 mg/kg doxycycline diet (Dox600), inguinal adipose tissue showed an increase in alcian blue staining, reflecting an increase of acidic polysaccharides in adipose tissue (Fig. 3E). Adipose tissue *Has2* overexpression increased tissue HA levels by approximately 10-fold (Fig. 3F). Adipose tissue-derived HA also entered circulation, leading to a 45% increase in circulating HA levels (Fig. 3G). Electrophoresis of extracted HA from *Has2* overexpressing adipose tissue demonstrated that HAS2 mainly drove the production of very high molecular weight HA, with a molecular weight close to 6000 kDa (Fig. 3H).

*Has2* overexpressing white adipocytes also display a morphology of multilocular lipid droplets (Fig. 3E), but this is independent of higher uncoupling protein 1 (*Ucp1)* expression (Fig. 3I). Brown adipose tissue *Ucp1* expression was not different either (Supplementary Fig. 2D). Notably, expression of mitochondrial creatine kinases *Ckmt2* is drastically increased in about half of mice (Supplementary Fig. 2E). Genes in other UCP1-independent thermogenesis processes, such as *Atp2a2* in calcium cycling and *Pm20d1* in n-acyl amino acid metabolism, did not changed (Supplementary Fig. 2F). Cytokine *Gdf15* expression was not different, while *Fgf21* expression was significantly decreased (Supplementary Fig. 2G). There was no change in collagen gene expression levels (Fig. 3I) but a decrease in genes involved in adipogenesis was apparent (Supplementary Fig. 2H). Metabolically, 5-day *Has2* overexpression in adipocytes has no effect on body weight (Supplementary Fig. 2I) or glucose tolerance (Supplementary Fig. 2J).

### Adipose Tissue *Has2* overexpression improves glucose tolerance on an HFHS diet

**Fig. 4:**
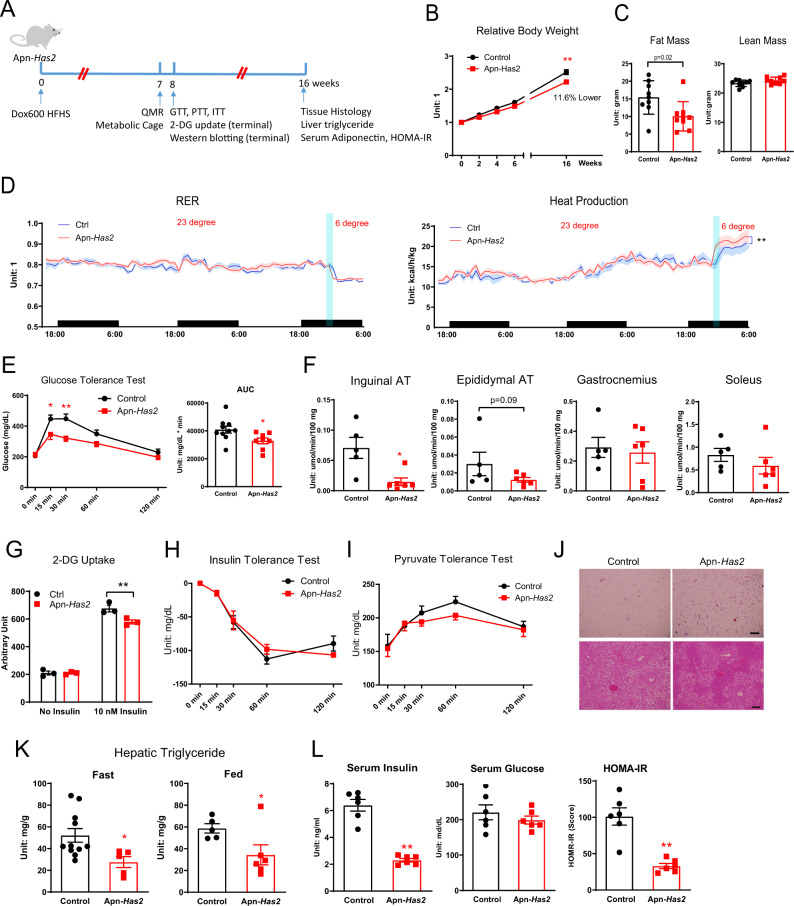
Adipose tissue *Has2* overexpression improves glucose tolerance on HFHS. **A** Schematic representation of Apn-*Has2* mouse treatment for panels **B**–**L**. Multiple cohorts of mice were used. **B** Relative body weights of Apn-*Has2* animals on Dox600 HFHS diet (*n* = 9 mice per each genotype for period 0–6 weeks, *n* = 6 mice per each genotype for body weight measured at 16 weeks). Two-way ANOVA followed by Sidak’s multiple comparisons test, adjusted *p*-value > 0.9999, 0.6705, 0.2137, 0.1131, <0.0001 for 0, 2, 4, 6, 16 weeks, respectively. **C** Fat mass and lean mass of Apn-*Has2* mice 7 weeks on Dox600 HFHS diet (*n* = 9 mice per each genotype). Two-tailed *t*-test. **D** RER and heat production of Apn-*Has2* mice measured in metabolic cages. Cage temperature was reduced from 23 degree to 6 degree in the middle of the third night. Heat production in control and Apn-*Has2* mice after temperature change was analyzed using two-way ANOVA. (*n* = 8 mice per each genotype). Two-way ANOVA test for time points after ramping to 6 °C (time points 66-75), *p* = 0.0008 for genotype factor, *p* = 0.0084 for time factor. **E** Glucose tolerance in Apn-*Has2* mice. (*n* = 10 mice for control, *n* = 8 mice for Apn-*Has2*). Two-way ANOVA followed by Sidak’s multiple comparisons test, adjusted *p*-value = 0.9994, 0.0174, 0.0014, 0.2834, 0.8711 for 0, 15, 30, 60, and 120 min, respectively. AUC was analyzed by two-tailed *t*-test, *p* = 0.0252. **F** 2-DG uptake in inguinal, epididymal adipose tissues (AT), and gastrocnemius and soleus muscles. (*n* = 5 mice for control, *n* = 6 mice for Apn-*Has2*). Two-tailed *t*-test, *p* = 0.0099, 0.0899, 0.7387, 0.3488, for inguinal, epididymal adipose tissues (AT), and gastrocnemius and soleus muscles, respectively. **G** Insulin-stimulated 2-DG uptake in differentiated adipocytes derived from SVF isolated from apn-*Has2* transgenic mice (*n* = 3 wells). Two-way ANOVA test, *p* < 0.0001 for treatment factor, *p* = 0.016 for genotype factor. Ctrl vs. Apn-*Has2*, *p* = 0.9995 (no insulin) and 0.0054 (10 nM insulin) for post-hoc Sidak’s multiple comparisons test. **H** Insulin tolerance in Apn-*Has2* mice (*n* = 3 mice for control, *n* = 4 mice for Apn-*Has2*). Two-way ANOVA test. **I** Pyruvate tolerance in Apn-*Has2* mice (*n* = 8 mice for control, *n* = 7 for Apn-*Has2*). Two-way ANOVA test. **J** Representative histology of inguinal adipose tissue (upper panels) and liver (lower panels) from Apn-*Has2* mice after 16 weeks Dox600 HFHS diet treatment. Scale bar = 100 µm. **K** Quantification of liver triglyceride from Apn-*Has2* mice under fast or fed conditions after 16 weeks Dox600 HFHS diet treatment. (Under fasting condition: *n* = 11 mice for control, *n* = 5 mice for Apn-*Has2*; under fed condition: *n* = 5 mice for control, *n* = 6 mice for Apn-*Has2*). Two-tailed *t*-test, *p* = 0.0286, and 0.0481 for fast and fed conditions, respectively. **L** Fasting insulin, fasting glucose, and HOMA-IR of Apn-*Has2* mice after 16 weeks Dox600 HFHS diet treatment. (*n* = 6 mice per genotype). Two-tailed *t*-test, *p* < 0.0001, =0.3828, and 0.0003 for fasting serum insulin, fasting glucose, and HOMA-IR, respectively. All data are presented as mean ± s.e.m. *indicates *p* ≤ 0.05, **indicates *p* ≤ 0.01.

The effect of HA on systemic glucose metabolism was first tested in mice with whole-body *Has2* (w-*Has2* TG) overexpression driven by R26-M2rtTA. After doxycycline-induced transgene expression (10 mg/kg in diet) and metabolic challenge by HFHS diet for 8 weeks, mice displayed a 9.5% lower body weight despite the same body weight at the starting point of the study (Supplementary Fig. 3A), and also showed an improved glucose tolerance (Supplementary Fig. 3B). Subsequently, a more in-depth metabolic characterization was carried out on adipose tissue-specific *Has2* overexpression mice (Apn-rtTA X TRE-*Has2*, abbreviated as Apn-*Has2*) mice (Fig. 4A). On Dox600 HFHS diet, body weight diverges early on: two weeks after initiation of diet treatment, Apn-*Has2* mice weigh 5.4% less, subsequently, the difference further expands to 7.3% after four weeks; after 16 weeks of an HFHS diet challenge, Apn-*Has2* mice are 11.7% lighter than the control mice on the same diet (Fig. 4B). Body composition measurements performed 7 weeks after initiation of dietary treatment revealed a 34.8% reduction in fat mass, with the same lean mass in Apn-*Has2* mice (Fig. 4C). When normalized to body weight, lean mass was in fact marginally increased (Supplementary Fig. 3C). Despite a reduction in expression of adipogenesis genes after 5 days of transgene induction, there was no difference in the PDGFRβ+ progenitor pool in the stromal vascular fraction of Apn-*Has2* mice fed with Dox600 HFHS diet for 8 weeks (Supplementary Fig. 3D). Mice with matched body weights were selected and used for a metabolic cage study. The results show similar levels of food intake, activity, energy expenditure and RER in Apn-*Has2* mice at room temperature (Fig. 4D and Supplementary Fig. 3E). However, when mice were exposed to a 6-h period in a cold environment (6 °C), Apn-*Has2* mice demonstrated enhance heat production (Fig. 4D).

Glucose tolerance was significantly improved in Apn-*Has2* mice after 8 weeks of dietary treatment (Fig. 4E). The improvements in glucose tolerance did not come from additional glucose uptake into the adipose tissue. In fact, 2-deoxyglucose (2-DG) uptake was significantly blunted in the inguinal fat depots (Fig. 4F). Similar trends were observed for the epididymal depot, whereas muscle glucose uptake was unaffected (Fig. 4F). These differences could also not be explained by circulating insulin levels (Supplementary Fig. 3F). In vitro differentiated adipocytes overexpressing *Has2* also displayed an impaired insulin-stimulated 2-DG uptake (Fig. 4G). Insulin tolerance tests reflect no improvements in peripheral insulin sensitivity in Apn-*Has2* mice (Fig. 4H). In contrast, pyruvate tolerance tests (which reflect hepatic gluconeogenesis), were slightly improved in Apn-*Has2* mice (Fig. 4I), suggesting a contribution of improved hepatic gluconeogenesis to improved whole-body glucose metabolism. Terminal analysis after 16 weeks of Dox600 HFHS treatment revealed Apn-*Has2* adipocytes were significantly smaller (Fig. 4J), hepatic triglyceride content was significantly lower (Fig. 4J, K), with no differences in serum ALT/AST (Supplementary Fig. 3G). Apn-*Has2* mice had an improved HOMA-IR score that was mostly contributed by reduced fasting insulin (Fig. 4L), while circulating adiponectin levels remained unchanged (Supplementary Fig. 3H).

### HA injection only marginally improves glucose tolerance in diet-induced obese mice

**Fig. 5:**
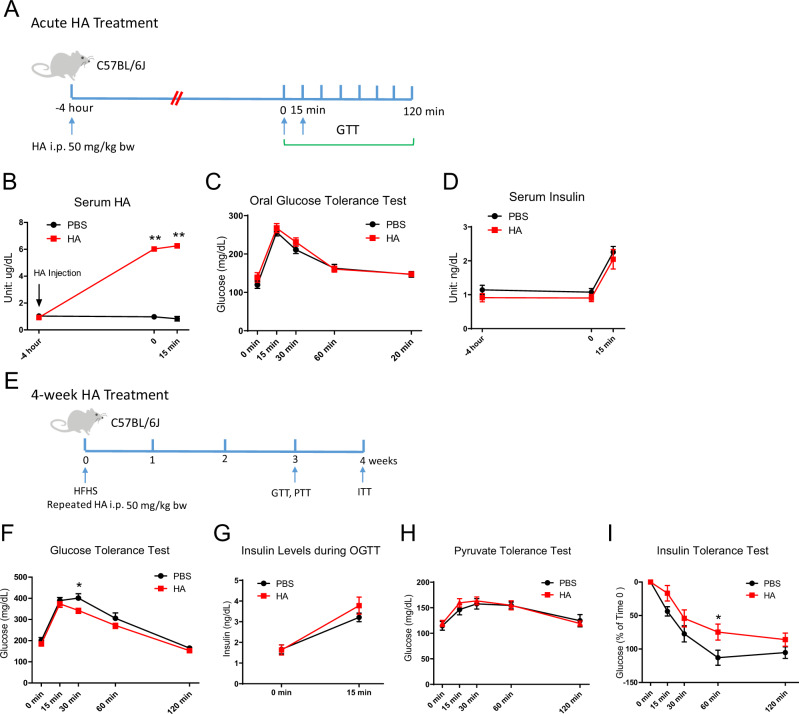
Effects of HA i.p. injections on glucose metabolism. **A** Schematic representation of mouse treatment for panels **B**–**D**. **B** Serum HA levels after an HA i.p. injection (*n* = 6 mice per group). Two-way ANOVA followed by Sidak’s multiple comparisons test, adjusted *p*-value = 0.8642, <0.0001, <0.0001 for -4 h, 0, and 15 min, respectively. **C** Oral glucose tolerance test 4 h after an HA i.p. injection (*n* = 12 mice per group). **D** Serum insulin levels assayed at times indicated by blue arrows in panel **A** (*n* = 12 mice per group). **E** Schematic representation of mouse treatment for panels **F**–**I**. Two cohorts of mice were used. **F** Repeated HA treatment effects on oral glucose tolerance (*n* = 8 mice per group). Two-way ANOVA followed by Sidak’s multiple comparisons test, adjusted *p*-value = 0.9546, 0.9700, 0.0467, 0.4970, 0.9873 for 0, 15, 30, 60, and 120 min, respectively. **G** Serum insulin levels after an oral glucose challenge (*n* = 4 mice for PBS, *n* = 6 mice for HA treatment). **H** Repeated HA treatment effects on pyruvate tolerance test (*n* = 8 mice for PBS, *n* = 9 mice for HA treatment). **I** Repeated HA treatment effects on insulin sensitivity. Glucose levels are plotted as the percentage of Time 0 (*n* = 8 mice). Two-way ANOVA followed by Sidak’s multiple comparisons test, adjusted *p*-value = 1, 0.2196, 0.3748, 0.0375, 0.6450 for 0, 15, 30, 60, and 120 min, respectively. All data are presented as mean ± s.e.m. *indicates *p* ≤ 0.05, **indicates *p* ≤ 0.01. Two-way ANOVA (**C**) (**D**) (**G**) (**H**).

Apn-*Has2* mice also have increased circulating HA levels, likely due to the release of HA from the enlarged adipose tissue HA pool. To understand whether circulating HA has a direct effect on other organs to improve glucose tolerance, we injected mice with HA intraperitoneally (i.p.) (50 mg/kg body weight) to increase circulating HA levels (Fig. 5A). An oral gavage of HA at the same dose failed to affect plasma HA levels (Supplementary Fig. 4A). Four hours after the injection, circulating HA levels were increased five-fold (Fig. 5B), but neither glucose tolerance (Fig. 5C) nor glucose-stimulated insulin secretion during the oral glucose tolerance test were different (Fig. 5D). This indicates that an acute increase in circulating HA does not modulate glucose homeostasis in a positive way.

We subsequently treated mice that were fed with an HFHS diet concurrently with the HA i.p. treatment on every second day for 4 weeks (Fig. 5E), which elevated circulating HA levels by at least 50% between two injections (Supplementary Fig. 4B). HA treatment had no effect on weight gain after 4 weeks (Supplementary Fig. 4C), nor did it affect food uptake (Supplementary Fig. 4D). Even three weeks of HA treatment only marginally improved glucose tolerance (Fig. 5F), with no differences in insulin levels 15 min post the oral glucose administration (Fig. 5G). Pyruvate tolerance tests did not differ between the HA treatment group and the PBS control group (Fig. 5H). The insulin tolerance test revealed a mild impairment in peripheral insulin sensitivity (Fig. 5I). Altogether this reflects a marginal effect of systemic HA treatment on glucose homeostasis and can’t account for metabolic improvement seen in Apn-*Has2* mice.

### Increasing circulating HA by hepatic *Has2* overexpression does not improve glucose tolerance

**Fig. 6:**
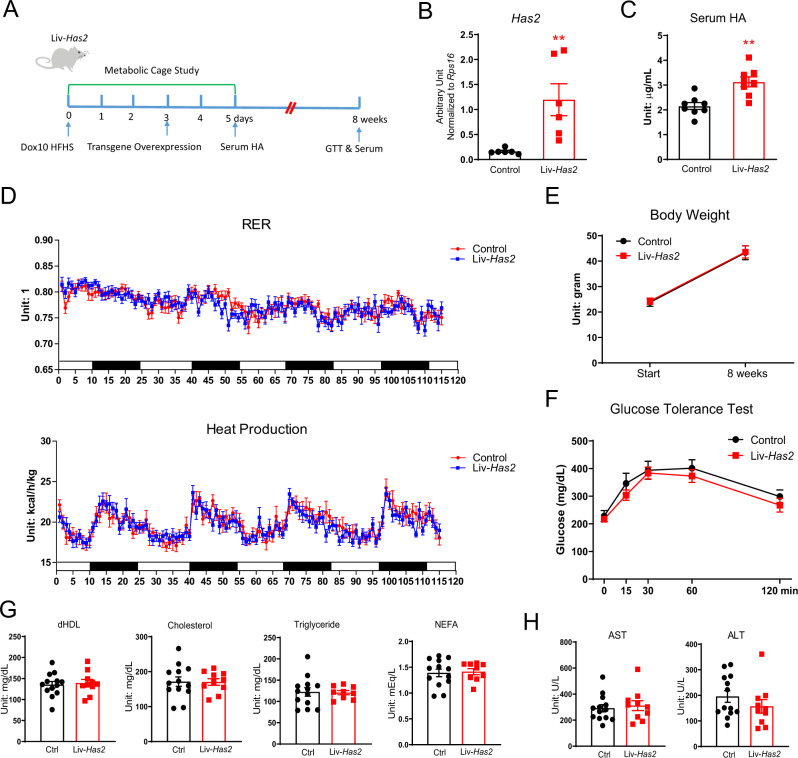
Hepatic *Has2* overexpression has no effect on glucose tolerance. **A** Schematic representation of Liv-*Has2* mouse treatment for panels **B**–**H**. Multiple cohorts of mice were used. **B** *Has2* gene expression in Liv-*Has2* livers on Dox10 HFHS diet for 3 days (*n* = 6 mice). Two-tailed *t*-test, *p* = 0.0013. **C** Serum HA for Liv-*Has2* mice on Dox10 HFHS diet for 5 days (*n* = 8 mice). Two-tailed *t*-test, *p* = 0.009. **D** RER and calculated heat production in Liv-*Has2* mice during 5-day of Dox10 HFHS diet feeding starting on the day 0 of metabolic cage study (*n* = 12 mice). **E** Body weight of Liv-*Has2* mice before and after Dox10 HFHS diet for 8 weeks (*n* = 6 mice for control, *n* = 7 mice for Liv-*Has2*). **F** Glucose tolerance test of Liv-*Has2* mice after 8 weeks of Dox10 HFHS treatment (*n* = 6 mice for control, *n* = 7 mice for Liv-*Has2*). **G** Serum lipids levels of Liv-*Has2* mice after 8 weeks of Dox10 HFHS treatment (*n* = 13 mice for control, *n* = 10 mice for Liv-*Has2*). **H** Serum AST and ALT levels of Liv-*Has2* mice after 8 weeks of Dox10 HFHS treatment. (*n* = 13 mice for control, *n* = 10 mice for Liv-*Has2*). All data are presented as mean ± s.e.m. **indicates *p* ≤ 0.01. Two-tailed *t*-test (**G**) (**H**); two-way ANOVA (**D**) (**E**) (**F**).

Injection of HA may cause a significant fluctuation in circulating HA not seen in mice, so we used another mouse model—a liver-specific, doxycycline-inducible *Has2* overexpressing mouse (Liv-*Has2*)—to understand the relationship between circulating HA and systemic glucose homeostasis (Fig. 6A). There is a dose-dependent effect of doxycycline on circulating HA levels after 5 days of diet treatment in this mouse model (Supplementary Fig. 5A). Higher doxycycline doses (200 mg/kg or 600 mg/kg in the diet) lead to the death of the mice within 3 days. We selected an HFHS diet containing 10 mg/kg doxycycline (Dox10 HFHS) as the dose for the subsequent metabolic characterization (Supplementary Fig. 5A). Gene expression analysis revealed a more than 7-fold increase in hepatic *Has2* expression (Fig. 6B) and 45% increase in serum HA levels (Fig. 6C). This increase of serum HA is on par with what can be obtained in Apn-*Has2* animals on Dox600 diets. A 5-day metabolic cage experiment, with diet treatment started after the metabolic cage study was initiated, showed no effects due to elevated circulating HA levels on body weight or body composition (Supplementary Fig. 5B). Food intake and water consumption did not differ either (Supplementary Fig. 5C). VO_2_ and VCO_2_ were not different (Supplementary Fig. 5D), nor were respiratory exchange ratios (RER) or heat production in Liv-*Has2* mice (Fig. 6D). After 8 weeks of Dox10 HFHS treatment, the mice had gained similar amounts of weight (Fig. 6E) and displayed the same level of glucose tolerance (Fig. 6F); none of the serum lipid profiles measured were different (Fig. 6G); hepatic function was not compromised, as indicated by unaltered AST and ALT levels (Fig. 6H).

When the doxycycline dose was raised to 50 mg/kg (Dox50), the circulating HA levels were further increased to two-fold above the levels of the control mice (Supplementary Fig. 5A). After a 4-week Dox50 HFHS treatment, mice gained less weight (Supplementary Fig. 5E), but serum lipids profiles and glucose tolerance remained the same (Supplementary Fig. 5F, G). With the liver AST and ALT levels trending up, together with a lethal phenotype of Liv-*Has2* mice on Dox200 diet, these data suggest potential liver toxicity upon higher level *Has2* overexpression in the liver (Supplementary Fig. 5H).

### Cellular *Has2* overexpression primes cells for lipolysis

**Fig. 7:**
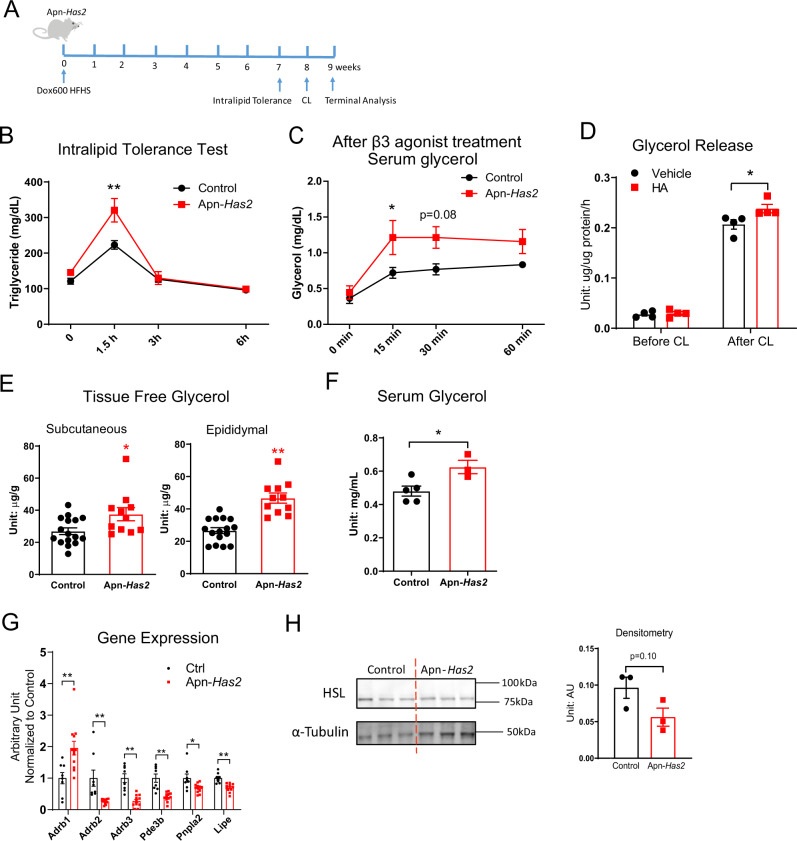
Cellular Has2 overexpression primes cells for lipolysis. **A** Schematic representation of Apn-*Has2* mouse treatment for panels **B**–**H**. Multiple cohorts of mice were used; CL: CL 316,243. **B** Intralipid tolerance test for Apn-*Has2* mice (*n* = 6 mice per genotype). Two-way ANOVA followed by Sidak’s multiple comparisons test, adjusted *p*-value = 0.7001, 0.0002, 0.9997, 1 for 0, 1.5, 3, and 6 h, respectively. **C** Glycerol release after β3 adrenergic receptor agonist CL 316,243 treatment (*n* = 5 mice per group). Two-way ANOVA followed by Sidak’s multiple comparisons test, adjusted *p*-value = 0.9869, 0.0439, 0.0813, 0.3049 for 0, 15, 30, and 60 min, respectively. **D** Glycerol release from in vitro differentiated adipocytes treated with HMW HA (10 µg/mL, 5 h) before and with CL 316,243 (1 µM) stimulation for 2 h (*n* = 4 mice per group). Two-way ANOVA followed by Sidak’s multiple comparisons test, adjusted *p*-value = 0.9749, 0.0125 for Vehicle vs. HA treatment before CL and after CL, respectively. **E** Tissue glycerol levels in Apn-*Has2* mice (*n* = 16 mice for control, *n* = 11 mice for Apn-*Has2*). Two-tailed *t*-test, *p* = 0.0187, <0.0001 for Subcutaneous and Epididymal depot, respectively. **F** Serum glycerol of Apn-*Has2* mice after overnight fasting (*n* = 5 mice for control, *n* = 3 mice for Apn-*Has2*). Two-tailed *t*-test, *p* = 0.0266. **G** Expression of lipolysis related genes in inguinal adipose tissue from mice treated with 5 days of Dox600 chow diet (*n* = 8 mice for control, *n* = 12 mice for Apn-*Has2*). Multiple two-tailed *t*-test, *p* = 0.0053, 0.0024, 0.00003, 0.00008, 0.014, 0.002 for *Adrb1, Adrb2, Adrb3, Pde3b, Pnpla2, Lipe*, respectively. **H** Western blot of HSL protein. Tissue lysates were prepared from the white adipose tissue dissected from mice treated with 9 weeks of Dox600 HFHS diet. HSL and Tubulin were blotted on two membranes in parallel. Densitometry result of HSL signal normalized to α-Tubulin is shown on the right (*n* = 3 mice per genotype). Two-tailed *t*-test, *p* = 0.10. All data are presented as mean ± s.e.m. *indicates *p* ≤ 0.05, **indicates *p* ≤ 0.01.

Adipose tissue *Has2* overexpression leads to a cellular morphology with multilocular lipid droplets in adipocytes (Fig. 3E). When mice were exposed to 16 weeks of HFHS feeding, the adipocytes appeared significantly smaller (Fig. 4J), consistent with a role of *Has2* expression in adipocyte lipid metabolism. To further understand how *Has2* affects adipocyte lipid metabolism, additional cohorts of Apn-*Has2* mice were assayed (Fig. 7A). After an oral gavage of intralipid, Apn-*Has2* mice pre-fed 7 weeks of Dox600 HFHS showed higher plasma triglycerides (Fig. 7B). Given that *Has2* overexpression is specific to the adipocytes, the intestinal lipid absorption is expected to be the same. A higher plasma triglyceride indicates an impaired clearance of circulating triglyceride in those mice. When Apn-*Has2* mice were challenged with CL316,243, a β3-adrenergic receptor agonist, they had more glycerol in circulation despite the same basal glycerol levels (Fig. 7C). Enhanced glycerol release upon CL316,243 treatment was also seen in in vitro differentiated adipocytes treated with high molecular weight HA (Fig. 7D). Despite the lack of a difference in circulating triglycerides or NEFA levels in *Apn-Has2* mice on both normal diet and HFHS diets (Supplementary Fig. 6A), free-glycerol levels from inguinal and epididymal fat depots of Apn-*Has2* mice were much higher (Fig. 7E). This also leads to higher fasting serum glycerol (Fig. 7F). The Apn-*Has2* inguinal depots also had higher levels of *Aqp3* and *Aqp9* transcripts, each encoding a water/glycerol transporter (Supplementary Fig. 6B). Expression of *Swell1*, a cellular volume sensor on the plasma membrane, is also increased in Apn-*Has2* inguinal depots (Supplementary Fig. 6B). Altogether these data indicate that adipose tissue *Has2* overexpression prompts adipocytes to generate more free glycerol, which is readily released upon stimulation and may inhibit clearance of triglyceride from the circulation.

However, Apn-*Has2* inguinal adipose tissue shows a decreased expression of lipolysis related genes (Fig. 7G) and reduced amounts of total HSL protein when normalized to α-Tubulin (Fig. 7H). Despite the enhanced lipolysis upon challenges like fasting, CL316,243 treatment or cold exposure, the livers of Apn*-Has2* mice displayed lower triglyceride content in both the fasting and the fed state (Fig. 4K), suggesting that the FFAs released from adipocytes due to enhanced adipose tissue lipolysis are effectively metabolized by the liver. Livers from mice treated for four weeks with HA showed an increase in ketone bodies (Supplementary Fig. 6C), reflecting an increase in hepatic fatty acid oxidation in response to elevated circulating HA.

Similarly, hepatic *Has2* overexpression led to reduced hepatic triglyceride content on HFHS diets (Supplementary Fig. 6D, E) despite an absence of any improvements in glucose tolerance, further indicating a role of HA in mobilizing lipid droplets.

## Discussion

An increase in HA has been implicated in the etiology of both Type 1^7^ and Type 2 diabetes^11^, and reducing HA by 4-MU treatment^7,27^ or administration of PEG-PH20^11^ were reported to exert beneficial effects, pointing to a pathological role of HA in developing both types of diabetes. However, as a natural phenomenon, naked mole-rats produce a much higher level of HA than laboratory rats, but live an extraordinarily long life without known metabolic abnormalities^28,29^. In the study reported here, we indeed observed an inverse correlation between circulating HA levels and overall metabolic fitness. However, to our surprise, several lines of evidence point to a beneficial effect of HA, especially in the adipose tissue. Whole-body *Has2* overexpression improves glucose tolerance, and this improvement is recapitulated with just adipose tissue *Has2* overexpression. Furthermore, we excluded the possibility of circulating HA as the major contributor of improved glucose tolerance.

A lot of interest has been centered around the use of 4-MU as an inhibitor of HA production to treat metabolic diseases. The excitement is partially due to the fact that 4-MU has been approved for clinical use in many countries as an over-the-counter choleretic and antispasmodic medication with a great safety profile. The compound has been demonstrated to improve the thermogenic capacity of brown adipose tissue^27^. However, our results raise the question whether 4-MU truly inhibits HA synthesis in adipose tissue, and where the metabolic benefits of long-term high-dose 4-MU treatment originate from. Firstly, in rodents, previous studies have demonstrated that the extraction rate of 4-MU by the gastrointestinal system to be around ~40% and by the liver to be almost ~100%^30^, suggesting the fraction of an oral administered 4-MU that reaches the systemic circulation as the original compound is very low. This pattern agrees with our observation that only intestinal HA levels were reduced by 4-MU treatment. Decreasing *Has2* levels were one of the proposed mechanisms for 4-MU to inhibit HA production. Lack of changes in *Has2* expression in the adipose tissue also suggests missing 4-MU actions in the adipose tissue. HFHS diet also did not increase expression of any of the *Has* isoforms (Fig. S2A). Secondly, the typically approved 4-MU dosing regimen for adults is 300–800 mg three times/day by mouth (i.e., 900–2400 mg/day). In contrast, the reported dose of 4-MU required in the literature to achieve a significant reduction in HA levels is much higher^24,25^. Mice fed chow containing 5% 4-MU, a dose calculated to deliver 250 mg/mouse/day, translates to 8 g/kg body weight in mice, far higher than the doses used in human subjects. This high dose of 4-MU treatment can lead to a transient repression of food intake, which may be the major contributor to the metabolic benefits seen. 4-MU used at such high doses could also have unfavorable effects on atherosclerosis^24^. Thirdly, another suggested major mechanism for 4-MU to inhibit HA production is via its function as a competitive substrate for UDP-glucuronosyltransferase (UGT) to synthesize UDP-N-acetylglucosamine (UDP-GlcNAc), one of the two substrates for HA synthesis^23^. UDP-GlcNAc is also extensively involved in intracellular signaling as a substrate for *O*-linked *N*-acetylglucosamine transferases, and it serves as a substrate for making glycosaminoglycans, proteoglycans, and glycolipids as well^31^. So, 4-MU is expected to affect a broad spectrum of cellular targets. Lastly, numerous reports have suggested a beneficial effect of HA in longevity^28^, joint health^13,14^ and protection from a septic response to LPS^32^ and colitis^33^. *HAS2* deficiency (reported only in a single person) leads to cardiac pathology, whereas a complete deficiency of *Has2* in mice causes embryonic lethality due to cardiac defects^34^, highlighting the importance of HA production in additional physiological processes not conventionally examined. HA is also essential for surfactant protein C-positive alveolar progenitor cell renewal^35^ and the maintenance of the endothelial glycocalyx^24,36^. All these observations strongly argue against using systemic inhibition of HA production to treat metabolic diseases. Despite those arguments, our study does not dispute the potential use of 4-MU for other diseases. Strategies taking advantage of the pharmacology of 4-MU of limited tissue exposure to target abnormal HA levels in the gastrointestinal track and the liver may still be viable therapeutic areas.

The beneficial effects of adipose tissue HA production on systemic glucose metabolism is likely multifactorial: using glucose to synthesize HA that then spills into circulation may dissipate a small amount of energy. It also reduces glucose uptake and primes adipocytes for lipolysis, preventing adipocytes from storing an excess amount of energy. This leads to reduced adipocyte size and improved overall metabolic health when other organs, including the liver, adapt and effectively handle excessive lipids that are conventionally handled by adipocytes. An increase in circulating ketone bodies after HA treatment suggests increased fatty acid oxidation and thus supports this hypothesis. Another possibility is that HA production in adipose tissue promotes beneficial remodeling, allowing enhanced secretion of favorable adipokines to communicate with other organs, such as the liver, to reduce gluconeogenesis.

A recent report described a self-assembled hyaluronic acid nanoparticle to be preferentially targeted to the adipose tissue, modulating adipose tissue inflammation, and alleviating insulin resistance^37^. This is consistent with our observations of beneficial effects of HA on adipose tissue proper. Whether inflammation plays a major role in our models will need to be investigated in future studies.

HA synthesis substrates GlcUA and GlcNAc are derived from glucose, and HA production in differentiated 3T3-L1 adipocytes is increased by high-glucose medium^38^, suggesting high circulating glucose levels in diabetes may lead to a systemic increase in HA levels. However, despite increased serum HA, adipose tissue from those obese mice showed a significant decrease in HA content (Supplementary Fig. 7A, B). This agrees with the lack of increase in adipose tissue *Has1, Has2, Has3* expression after HFHS diet treatment. Similarly, a disconnect of blood glucose levels and tissue HA levels was seen in Type 1 diabetes, promoting authors to suspect inflammation rather than glucose drives HA levels in diabetes^8^.

One interesting observation is the link between HA production and cellular lipolysis. We believe osmotic pressure may be the link between two phenomena, as indicated by changes in the cellular volume sensor *Swell1*^39^ upon *Has2* overexpression in adipocytes. We have discussed previously in a review^38^ that if the concentration of HA increases by two-fold, the osmotic pressure will increase by more than a four-fold. The osmotic pressure can be further increased if HA is connected to collagen, which is the case in WAT, where the pericellular HA is connected with Col VI. To counter the increased osmotic pressure, cells can accumulate polyols^40^, which, in the case of adipocytes, are represented by glycerol. The glycerol can be derived from lipolysis or de novo synthesis. However, the expression of several key enzymes in lipolysis was reduced, and HSL protein levels were also reduced in Apn-*Has2* adipose tissue, suggesting a mechanism independent of HSL in linking osmotic pressure to cellular glycerol accumulation.

One limitation of the study is that we could not systemically evaluate the size distribution of HA in all different models and different tissues. Size-dependent differences in hyaluronan signaling and downstream biological effects are well established^41^. However, it is inherently low-throughput and technically challenging to separate HA by molecular weight.

In summary, HA is a naturally occurring, critical component of the extracellular matrix. It displays great variation of abundance in different tissues, highlighting probable diverse functions in these tissues. HA in adipose tissue governs its biology and physiology, fundamentally linked to glucose and lipid metabolism. Strategies to target HA abundance and metabolism should have a global view and balance the benefit against adverse effects on other organs. Future work will need to look into the role of HA on other aspects of adipose tissue physiology, such as inflammation and adipogenesis. Determination of adipose tissue HA sizes under metabolic stress will also significantly deepen our understanding of the HA, as HA of different molecular weights likely engage distinct signaling events and play different roles.

## Methods

### Animals

Animals were cared and used in accordance with the United States Animal Welfare Act and experimental protocols were approved by the Institutional Animal Care and Use Committee of the University of Texas Southwestern Medical Center (UTSW) and Baylor College of Medicine. Adiponectin-rtTA mice were previously generated and verified in the lab^26^. TRE-*Has2* mice were generated by subcloning the mouse *Has2* gene into the pTRE vector (Clontech) with a rabbit β-globin 3’ UTR^42^. Systemic expressed R26-M2rtTA (The Jackson Laboratory, B6.Cg-Gt(ROSA)26Sortm1(rtTA*M2)Jae/J, Strain: 006965), liver-specific albumin-Cre transgenic (The Jackson Laboratory, B6.Cg-Speer6-ps1Tg(Alb-cre)21Mgn/J, Stock No: 003574) and Rosa26-loxP-STOP-loxP-rtTA transgenic (The Jackson Laboratory, Gt(ROSA)26Sortm1(rtTA,EGFP)Nagy/J, Stock No: 005572) mice were purchased from Jackson Laboratories. High-fat high-sucrose (HFHS) diet (S1850), HFHS containing doxycycline 10 mg/kg (S7341), 50 mg/kg (S7624) or 600 mg/kg (S7067) were purchased from Bio-Serv. 4-Methylumbelliferone (4-MU) was purchased from Sigma (M1381) and blended into HFHS diet in house at the indicated concentrations. Hyaluronic acid sodium salt from rooster comb (Sigma Aldrich, Ca. Number: H5388) was dissolved in sterile saline and administered to mice by indicated routes. All treatments were started at 8-10 weeks of age. Only male mice were used. Serum HA levels were measured using an ELISA kit (R&D systems, Cat. Number: DHYAL0) using manufacturer provided protocol, which detects all HA with a molecular weight higher than 35 KDa. Body composition was determined by quantitative magnetic resonance. Mice were randomly allocated to experimental groups, were weight-matched at the beginning of experimental protocols. Investigators were not blinded to treatment groups during studies.

### Studies conducted in human subjects

A total of 30 males and females with obesity participated in this study, which was conducted in the Clinical Translational Research Unit (CTRU) at Washington University School of Medicine in St. Louis, MO. All subjects completed a screening history and physical examination, standard blood tests and an75 g oral glucose tolerance test. Written informed consent was obtained from all subjects before their participation in this study, which was approved by the Institutional Review Board of Washington University School of Medicine in St. Louis, MO and registered in Clinical Trials.gov (NCT02706262). Participants were separated into two distinct groups: (i) obese with normal oral glucose tolerance (Obese-normal) (fasting plasma glucose concentration <100 mg/dL, 2-h OGTT plasma glucose concentration <140 mg/dL and HbA1c ≤5.6%); and (ii) obese with prediabetes (Obese-prediabetes) (fasting plasma glucose concentration ≥100 mg/dL 2-h OGTT plasma glucose concentration ≥140 mg/dL, and/or HbA1c ≥5.7%). On a separate occasion, subjects were admitted to the CTRU at 17:00 h on day 1 and consumed a standard meal between 18:00 h and 19:00 h. At 06:30 h the next morning on day 2, a catheter was inserted into an antecubital vein for serial blood sampling. At 07:00 h a meal was provided containing one-third of the participant’s energy requirements^17^ and was comprised of 50% carbohydrate, 35% fat, and 15% protein. Blood samples were obtained before and serially for 5 h after ingesting a mixed meal ingestion.

### Metabolic phenotyping

Glucose tolerance test (GTT), pyruvate tolerance test (PTT), insulin tolerance test (ITT) and Intralipid tolerance test were performed as previously described^43–46^. Serum insulin levels were measured using ALPCO Mouse Insulin ELISA Jumbo kit (Cat. Number: 80-INSMS-E10). β3 adrenergic receptor agonist CL 316,243 (Sigma, Ca. Number: C5976) was used at 1 mg/kg body weight through intraperitoneal injection, CL 316,243-stimulated glycerol release was performed as previously described^44^. Liver triglyceride extraction and quantification was performed at UTSW metabolic phenotyping core using a previously published protocol^43^. Serum parameters (aspartate transaminase, alanine transaminase, cholesterol, triglyceride, very low-density lipoprotein, low-density protein and direct high density lipoprotein) were measured and calculated with a VITROS analyzer (Ortho Clinical Diagnostics) at UTSW metabolic phenotyping core.

### Quantitative RT- qPCR

RNA was isolated from frozen tissues by homogenization in Trizol Reagent (Invitrogen) with the manufacturer provided protocol^47^. One microgram of RNA was used to transcribe cDNA using a reverse transcription kit (Bio-rad). RT–qPCR primers are obtained from Harvard PrimerBank^48^ and listed in Supplementary Table 1. The messenger RNA levels were calculated using the comparative threshold cycle (Ct) method, normalized to gene *Rps16* or *Rps18*.

### Western blotting

Protein extractions were performed as previously described^44,49^. Primary antibodies HAS2 (Santa Cruz Biotechnology, sc-34068) (1:200 dilution), HSL (Santa Cruz Biotechnology, sc-74489) (1:200 dilution), α Tubulin (Santa Cruz Biotechnology, sc-53030) (1:200 dilution), Actin (Sigma #A4700) (1:1000 dilution), Adiponectin (homemade) (1:1000 dilution) were used. Protein abundance was detected using the one of the following secondary antibodies: Goat anti-Mouse IRDye 680RD (Li-cor 926-68070), Goat anti-rabbit IRDye 800CW (Li-cor 925-32211) or Goat anti-Rat DyLight 800 (Thermo Fisher SA5-10024) at 1:10,000 dilutions. Antibody decorated membranes were then visualized on a Li-Cor Odyssey infrared scanner (Li-Cor Bioscience). The scanned data were analyzed using Odyssey Version 3.0 software (Li-Cor Bioscience).

### 2-deoxyglucose (2-DG) uptake in animals

C14 labeled 2-deoxyglucose (13 (μCi/mouse) was administered after a 6-h fast by tail-vein injection. After 25 min, tissues were rapidly dissected and frozen. The uptake of 2-deoxyglucose was calculated as previously described^50^.

### Insulin-stimulated 2-deoxyglucose (2-DG) uptake in differentiated adipocytes

Insulin-stimulated 2-DG uptake assays were performed in triplicate in 12-well culture plates modified from previous publications^47,51^. In brief, SVF was isolated, cultured and differentiated as described previously^52^. In all, 0.5 µg/mL doxycycline was added 1 day before to induce transgene expression. Differentiated adipocytes with transgene induction were conditioned in differentiation medium without insulin and doxycycline overnight before being fasted for 40 min in DMEM/F12 no glucose medium supplemented with 1 mg/mL BSA and 1 mM pyruvate. Glucose uptake was performed by adding 2-[1,2-3 H]-deoxy-D-glucose in the presence of 0 and 10 nM insulin. Relative uptake is calculated by quantifying the radioactivity of 2-[1,2-3 H]-deoxy-D-glucose-6-phosphate.

### Histology and imaging

Adipose tissue and livers were excised and fixed overnight in 10% PBS-buffered formalin and were thereafter stored in 50% ethanol. Tissues were sectioned (5 µm), rehydrated and stained at Pathology Core at UT Southwestern. Microscopic images were taken on a Keyence BZ-X710 imaging system.

### Flow cytometry

Minced Inguinal fat depots were digested for 2 h while shaking at 37 °C in buffer containing 100 mM HEPES (pH 7.4), 120 mM NaCl, 50 mM KCl, 5 mM glucose, 1 m CaCl_2_, 1.5% BSA, and 1 mg/mL collagenase D (Roche 11088882001). Digested tissues were then filtered through a 100 µm cell strainer and then centrifuged for 5 min at 600 × *g* to pellet the stromal vascular fraction (SVF) cells. Red blood cells were lysed using commercial lysis buffer (Sigma R7757). 1 × 10^6^ SVF cells were first incubated on ice for 20 min in 200 µl of 2% FBS/PBS containing anti-mouse CD16/CD32 Fc block (clone 2.4G2) at a dilution 1:200. Cells were then incubated with primary antibody (PE anti-CD31 clone 390 1:200; PE anti-CD45 clone 30-F11 1:200 and APC anti-CD140B clone APB5 3:200) and rotated at 4 °C for 30 min in the dark. Cells were then washed three times with 2% FBS/PBS at 600 × *g* for 5 min. Cells were analyzed using a FACS CantoIITM flow cytometer (UT Southwestern Medical Center Flow Cytometry Core Facility). Flow cytometry plots were generated with FlowJo Version 10.2. All antibodies were obtained commercially from BioLegend (San Diego, CA USA).

For analysis, cells were initially selected by size, on the basis of forward scatter (FSC) and side scatter (SSC). SV cells isolated from wild-type mice, along with fluorescent-minus-one (FMO) controls, were used to determine background fluorescence levels. The populations were gated on PE (CD31 and CD45) and APC (CD140B) channels. Percentages of Pdgfrβ+/Lin- cells were obtained from control and transgenic samples and quantified.

### HA extraction for ELISA

The procedure was performed as previously described^28^, extracted HA was quantified using an ELISA kit (R&D systems, Cat. Number: DHYAL0). The tissue HA content is normalized to the wet weight used for extraction.

### HA molecular weight (MW) electrophoresis

The procedure was performed as previously described with some modifications^53,54^. Following dissection of fat tissues and measuring wet weight, fat tissues were digested in 100 mM ammonium acetate with 0.0005% phenol red (pH 7.0) containing 0.25 mg/mL proteinase-K (Roche) for 4 h at 60 °C. Proteinase-K was inactivated by boiling, and undigested tissues were pelleted by centrifugation. An aliquot of 20 μL supernatant equal to 100 mg wet weight was then treated with 1 μL of deoxyribonuclease (Ambion, Austin, TX) and 1 μL of ribonuclease (Roche) for 2 h at 37 °C. Samples were boiled to inactivate enzymes, and were precipitated in 80% ethanol at −20 °C overnight. After centrifugation at 13,000 × *g*, pellets were resuspended in 20 μL of Tris-Na Acetate-EDTA (pH.7.9) and 3 μL loading buffer (0.2% Bromophenol Blue, 1 mL TAE buffer, and 8.5 mL glycerol). Samples were run on a 1% agarose gel (Seakem HGT; Cambrex, Rockland, ME) made in TAE buffer. The gel was pre-run for approximately 1 h at 100 V before loading samples and HA size standards (Hyalose, Oklahoma City, OK). After electrophoresis at 100 V, the gel was incubated in water with gentle shaking for 1 h, followed by incubation in Stains-All solution 2.5 mg/mL (Sigma) of 30% ethanol overnight in the dark. Gel was destained in water until bands were visualized before scanning.

### Adipocyte culture and CL 316,243 treatment

SVF was isolated, cultured and differentiated as described previously^52^. Culture medium with 10 µg/mL HA was added, 5 h after the treatment, culture medium was sampled, and refreshed with new medium containing 1 µM of CL 316,243, culture medium was sampled again, and cells were lysed to measure protein content. Cellular glycerol release was calculated by subtracting beginning medium glycerol content from the end glycerol content, and was normalized to the protein content.

### Free-glycerol extraction

Frozen accurately weighed adipose tissue samples (200 mg) were homogenized in 1 mL of MeOH in borosilicate glass tubes using a mechanical homogenizer. The homogenizer probe was rinsed with 1 mL of MeOH and the methanolic extracts were combined. 4 mL of Hexane was added to the methanolic homogenate, then samples were thoroughly vortexed for 30 s. Samples were then centrifuged in a bucket benchtop centrifuge at 2500 × *g* for 10 min. The hexane top layer was discarded and the methanolic bottom layer was re-extracted with additional 4 mL of hexane. The final bottom methanolic layer was dried under purified nitrogen stream without heat. Samples were reconstituted in 200 µL of ultrapure water. Twenty microliters of sample were used for analysis using the free-glycerol reagent from Sigma Aldrich (Catalog Number F6428).

### Metabolic cage experiment

Metabolic cage studies were conducted using a PhenoMaster System (TSE systems) at UTSW metabolic phenotyping core as previously described^55^. Mice were acclimated in temporary holding cages for 5 days before recording. Food intake, movement, and CO_2_ and O_2_ levels were measured at various intervals (determined by collectively how many cages were running concurrently) for the indicated period shown on figures.

### Statistics and reproducibility

All values are expressed as the mean ± s.e.m. *indicates *p* ≤ 0.05, **indicates *p* ≤ 0.01, unless the values were explicitly labeled on the graph. Micrographs in Figs. 2e; 3c, e; 4j and Supplementary Figs. 3h; 6d were a representative from at least three experiments.

### Reporting summary

Further information on research design is available in the Nature Research Reporting Summary linked to this article.

## Supplementary information

Supplementary Info

Reporting Summary

## Data Availability

All other relevant data are available from the corresponding authors upon reasonable request.
