# Supplementary material for: Adipose tissue hyaluronan production improves systemic glucose homeostasis and primes adipocytes for CL 316,243-stimulated lipolysis
Source: Nat Commun. 2021 Aug 10;12:4829. doi: 10.1038/s41467-021-25025-4 (PMC8355239; doi:10.1038/s41467-021-25025-4)
Supplement: Supplementary file 1 — Supplementary Info [file 41467_2021_25025_MOESM1_ESM.pdf]

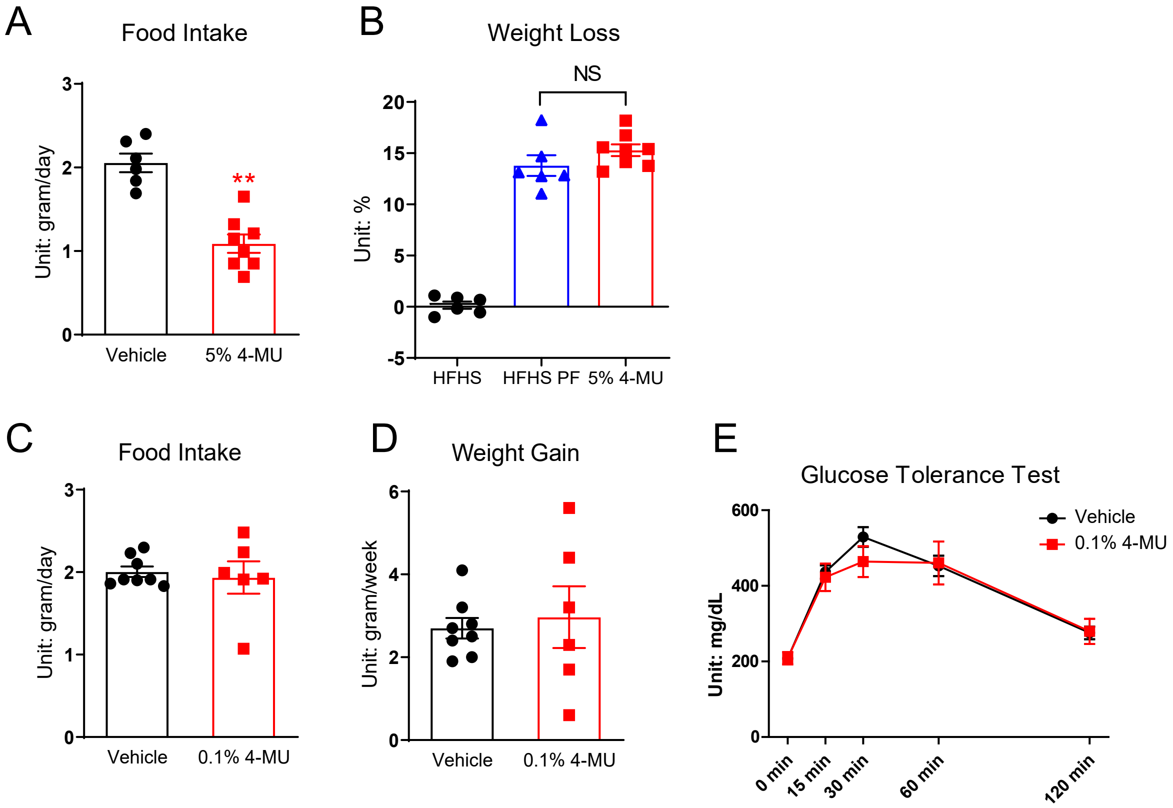

A
Food Intake
Unit: gram/day
3
2
1
0
**
Vehicle
5% 4-MU
B
Weight Loss
NS
Unit: %
20
15
10
5
0
-5
HFHS
HFHS PF
5% 4-MU
C
Food Intake
Unit: gram/day
3
2
1
0
Vehicle
0.1% 4-MU
D
Weight Gain
Unit: gram/week
6
4
2
0
Vehicle
0.1% 4-MU
E
Glucose Tolerance Test
Unit: mg/dL
600
400
200
0
Vehicle
0.1% 4-MU
0 min
15 min
30 min
60 min
120 min

Supplementary Fig. 1: 4-MU treatment on metabolic fitness

A. 4-MU HFHS diet treatment suppresses food intake after diet switch (n = 6 mice for control, n = 8 mice for 4-MU treatment group). Two-tailed t-test, $p < 0.0001$.

B. Body weight loss during 1-week 4-MU pair feeding regimen (n = 6 mice for control, n = 6 mice for pair-fed, n=8 for 4-MU treatment group). One-way ANOVA followed by Bonferroni's multiple comparisons test.

C. Food intake in mice treated with 0.1% 4-MU HFHS diet (n = 8 mice for control, n = 6 mice for 4-MU treatment group).

D. Body weight gain in mice treated with 0.1% 4-MU HFHS diet for two weeks (n = 8 mice for control, n = 6 mice for 4-MU treatment group).

E. Glucose tolerance in mice treated with 0.1% 4-MU HFHS diet for two weeks (n = 8 mice for control, n = 6 mice for 4-MU treatment group)

All data are presented as Mean ± s.e.m. ** indicates $p \leq 0.01$. Two-tailed *t*-test (C) (D). Two-way ANOVA (E).

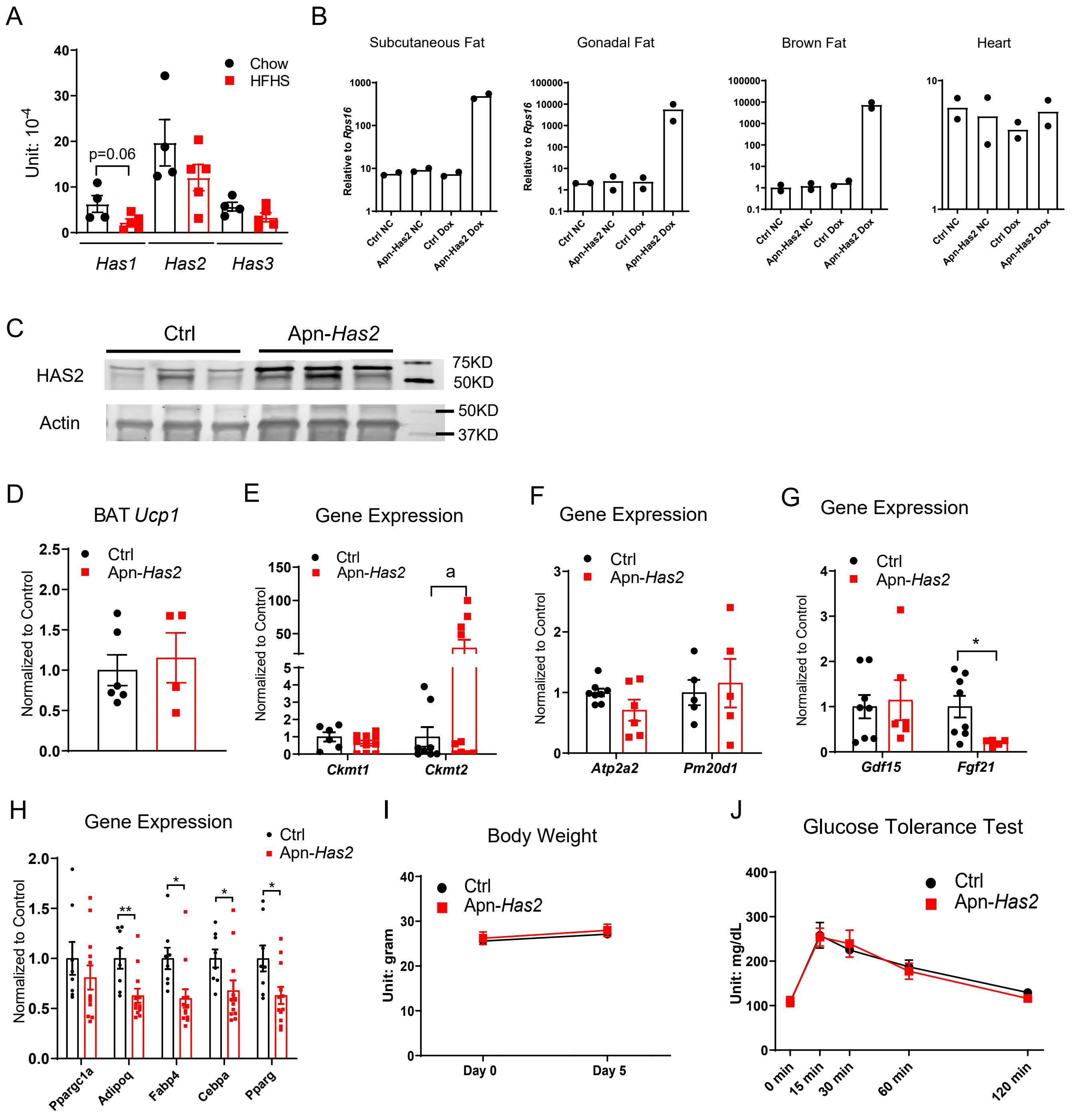
A
Unit: 10^-4
Chow
HFHS
p=0.06
Has1
Has2
Has3
B
Subcutaneous Fat
Gonadal Fat
Brown Fat
Heart
Relative to Rps16
Ctrl NC
Apn-Has2 NC
Ctrl Dox
Apn-Has2 Dox
C
Ctrl
Apn-Has2
HAS2
Actin
75KD
50KD
50KD
37KD
D
BAT Ucp1
Ctrl
Apn-Has2
Normalized to Control
E
Gene Expression
a
Ckmt1
Ckmt2
F
Gene Expression
Atp2a2
Pm20d1
G
Gene Expression
*
Gdf15
Fgf21
H
Gene Expression
**
*
Ppargc1a
Adipoq
Fabp4
Cebpa
Pparg
I
Body Weight
Unit: gram
Day 0
Day 5
J
Glucose Tolerance Test
Unit: mg/dL
0 min
15 min
30 min
60 min
120 min

Supplementary Fig. 2: Verification and characterization of Tre-*Has2* animals

A. *Has1, Has2* and *Has3* expression levels in the adipose tissue on normal chow (NC) or 8 weeks of HFHS diet (n = 4 mice for NC, 5 mice for HFHS treatment). Multiple two-tailed *t*-test, $p = 0.0631, 0.2101, 0.1373$ for *Has1, Has2* and *Has3,* respectively.

B. *Has2* expression in subcutaneous, gonadal and brown adipose tissues, as well as in the heart after 5-day Dox600 chow treatment (n = 2 mice).

C. HAS2 protein levels in Apn-*Has2* inguinal adipose tissue (n = 3 mice).

D. Expression of *Ucp1* in Apn-*Has2* brown adipose tissue (BAT) after 5-day Dox600 chow treatment (n = 6 mice for control, n = 4 mice for Apn-*Has2* mice).

E. Expression of *Ckmt1* and *Ckmt2* in Apn-*Has2* inguinal adipose tissue after 5-day Dox600 chow treatment (n = 8 mice for control, n = 10 mice for Apn-*Has2* mice). For *Ckmt1*, two samples in Ctrl group were not detected. Two-tailed *t*-test. a: $p = 0.051$.

F. Expression of *Atp2a2* and *Pm20d1* in Apn-*Has2* inguinal adipose tissue after 8-week Dox600 HFHS diet treatment (n = 8 mice for control, n = 6 mice for Apn-*Has2* mice). For *Pm20d1*, three samples in Ctrl group, one sample in Apn-*Has2* group were not detected.

G. Expression of *Gdf15* and *Fgf21* in Apn-*Has2* inguinal adipose tissue after 8-week Dox600 HFHS diet treatment (n = 8 mice for control, n = 6 mice for Apn-*Has2* mice). For *Fgf21*, one sample in Apn-*Has2* group were not detected. Multiple two-tailed *t*-test, p = 0.7699, 0.0256 for *Gdf15* and *Fgf21*, respectively.

H. Expression of adipogenesis related gene in Apn-*Has2* inguinal adipose tissue after 5-day Dox600 chow treatment (n = 8 mice for control, n = 12 mice for Apn-*Has2* mice). Multiple two-tailed *t*-test, $p = 0.3557, 0.0064, 0.0123, 0.0405, 0.0228$ for *Ppargc1a, Adipoq, Fabp4, Cebpa, and Pparg,* respectively.

I: Body weights after 5-day Dox600 chow diet treatment (n = 4 mice per genotype).

J: Glucose tolerance after 5-day Dox600 chow diet treatment (n = 4 mice per genotype).

All data are presented as Mean ± s.e.m. * indicates $p \leqslant 0.05$, ** indicates $p \leqslant 0.01$. Two-tailed *t*-test (D). Multiple *t*-test (F). Two-way ANOVA (I) (J).

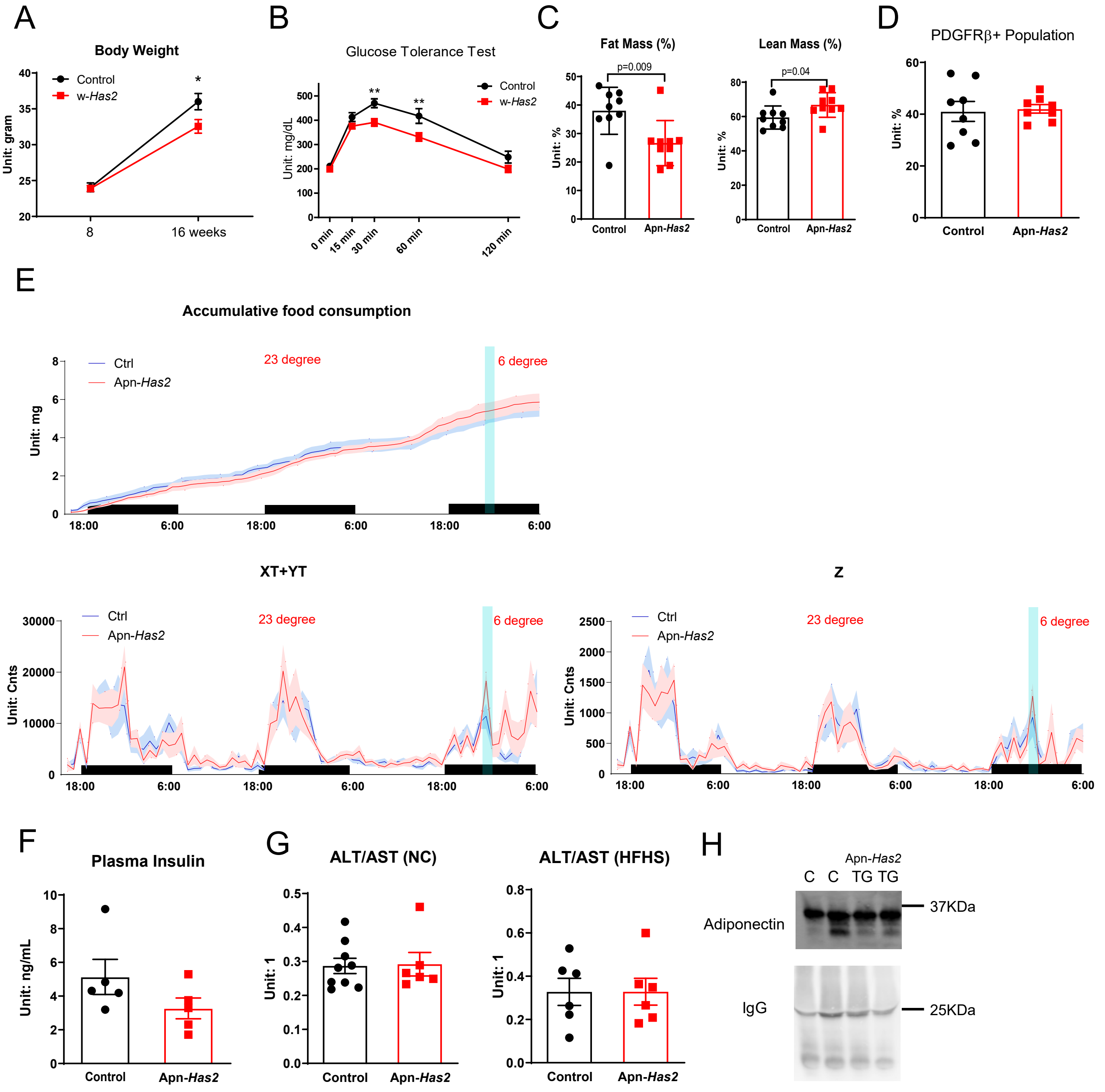
A
Body Weight
Unit: gram
Control
w-Has2
*
8
16 weeks
B
Glucose Tolerance Test
Unit: mg/dL
Control
w-Has2
**
**
0 min
15 min
30 min
60 min
120 min
C
Fat Mass (%)
p=0.009
Unit: %
Control
Apn-Has2
Lean Mass (%)
p=0.04
Unit: %
Control
Apn-Has2
D
PDGFRβ+ Population
Unit: %
Control
Apn-Has2
E
Accumulative food consumption
Unit: mg
Ctrl
Apn-Has2
23 degree
6 degree
18:00
6:00
18:00
6:00
18:00
6:00
XT+YT
Unit: Cnts
Ctrl
Apn-Has2
23 degree
6 degree
Z
Unit: Cnts
Ctrl
Apn-Has2
23 degree
6 degree
F
Plasma Insulin
Unit: ng/mL
Control
Apn-Has2
G
ALT/AST (NC)
Unit: 1
Control
Apn-Has2
ALT/AST (HFHS)
Unit: 1
Control
Apn-Has2
H
Apn-Has2
C
C
TG
TG
Adiponectin
37KDa
IgG
25KDa

Supplementary Fig. 3: Effects of *Has2* overexpression on metabolic profile

A: Starting and terminal body weight of w-*Has2* mice after 8 weeks of Dox10 HFHS diet treatment (n = 8 mice for control, n = 11 mice for w-*Has2* mice). 2way ANOVA followed by Sidak's multiple comparisons test, control vs. w-Has2, adjusted p = 0.9897 and 0.0115 for Before and After, respectively.

B: Glucose tolerance test for w-*Has2* mice after 8 weeks of Dox10 HFHS diet treatment (n = 8 mice for control, n = 11 mice for w-*Has2* mice). 2way ANOVA followed by Sidak's multiple comparisons test, adjusted p value = 0.9914, 0.4654, 0.0063, 0.0022, 0.1910 for 0 min, 15 min, 30 min, 60 min and 120 min, respectively.

C: Fat mass and lean mass expressed as percentage of body weight of Apn-*Has2* mice after 7 weeks Dox600 HFHS treatment (n = 9 mice per genotype). Two-tailed *t*-test.

D: Adipocyte PDGFRβ+ progenitor population in Apn-*Has2* inguinal adipose stromal vascular fraction after 8 weeks of Dox600 HFHS treatment (n = 8 mice per genotype).

E: Cumulative food consumption, XT+YT activity and Z activity measured in metabolic cage experiment (n = 8 mice per genotype).

F: 4-h fasting insulin levels between control and Apn-*Has2* mice after 8 weeks of Dox600 HFHS treatment (n = 5 mice per genotype).

G: Serum ALT/AST ratio from Apn-*Has2* on Dox600 chow diet (NC) or Dox600 HFHS diet (HFHS) for 16 weeks (under NC: n = 9 mice for control, n = 6 mice for Apn-*Has2*; under HFHS: n = 6 mice for control, n = 6 mice for Apn-*Has2*).

H: Representative blot showing serum adiponectin levels in control and Apn-*Has2* mice after 16 weeks Dox600 HFHS treatment.

All data are presented as Mean ± s.e.m. * indicates $p \leqslant 0.05$, ** indicates $p \leqslant 0.01$. Two-tailed *t*-test (D) (F) (G). Two-way ANOVA (E).

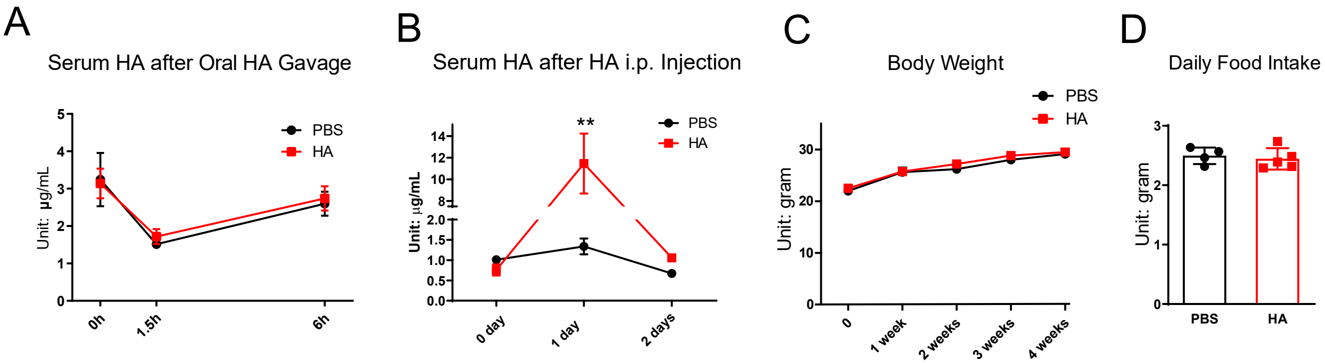

A
Serum HA after Oral HA Gavage
PBS
HA
Unit: µg/mL
0h
1.5h
6h
B
Serum HA after HA i.p. Injection
**
PBS
HA
Unit: µg/mL
0 day
1 day
2 days
C
Body Weight
PBS
HA
Unit: gram
0
1 week
2 weeks
3 weeks
4 weeks
D
Daily Food Intake
Unit: gram
PBS
HA

Supplementary Fig. 4: Effects of HA treatment of mice

A. Serum HA levels after HA oral gavage (n = 4 mice per treatment).

B: Serum HA levels 2 days after an i.p. injection of HA (50 mg/kg body weight) (n = 4 mice per treatment). 2way ANOVA followed by Sidak's multiple comparisons test, adjusted *p* value = 0.9981, <0.0001, 0.9935 for 0 day, 1 day, 2 days, respectively.

C: Body weight during 4 weeks of HA treatment. Mice were treated with HFHS diet concurrently (n = 8 mice per treatment).

D: Food intake after initiation of the 4-week HA treatment regimen (n = 4 mice for PBS, n = 5 mice for HA treatment).

All data are presented as Mean ± s.e.m. ** indicates $p \leq 0.01$. Two-tailed *t*-test (D). Two-way ANOVA (A) (C).

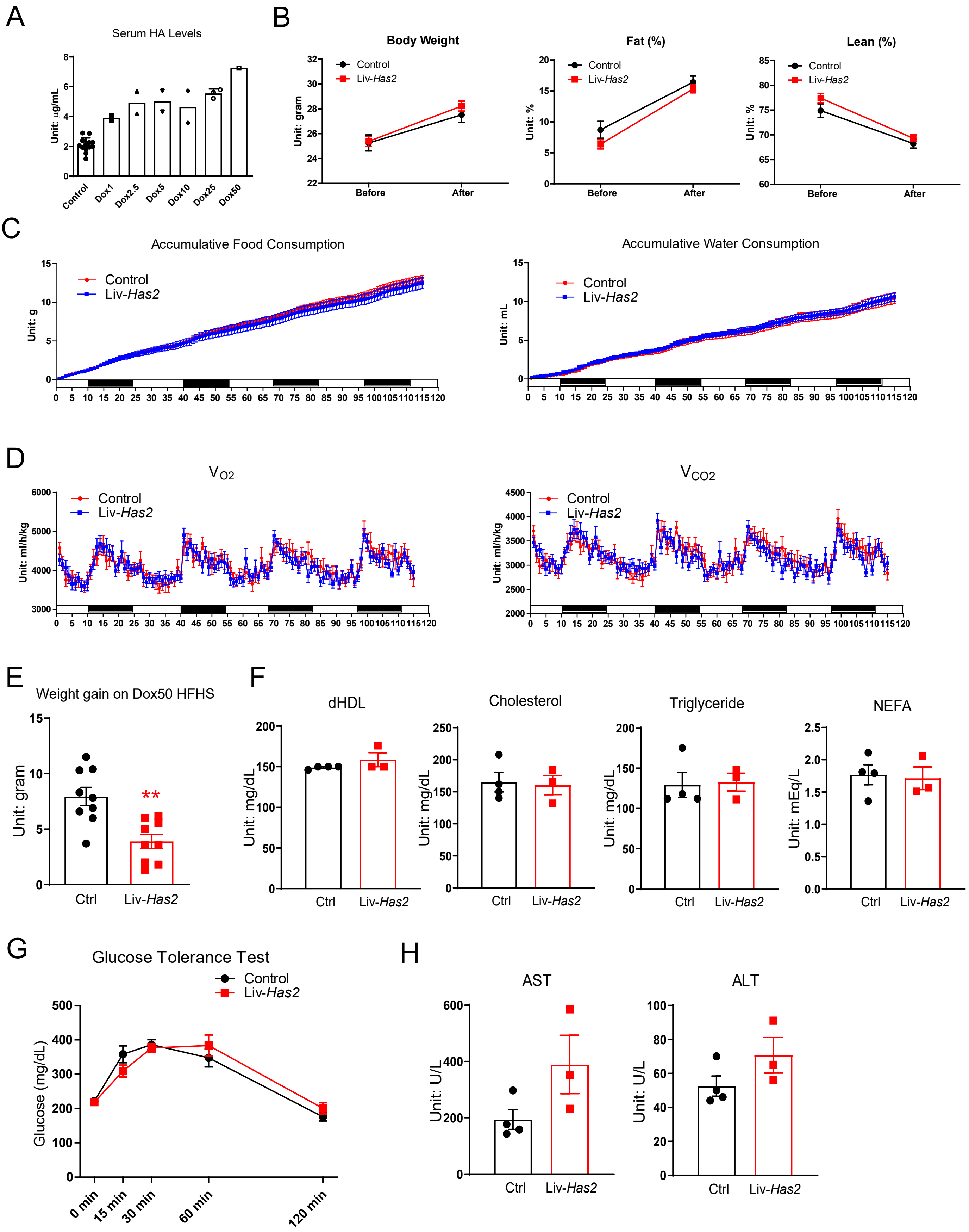
A
Serum HA Levels
Unit: µg/mL
Control
Dox1
Dox2.5
Dox5
Dox10
Dox25
Dox50
B
Body Weight
Unit: gram
Fat (%)
Unit: %
Lean (%)
Unit: %
Control
Liv-*Has2*
Before
After
C
Accumulative Food Consumption
Unit: g
Accumulative Water Consumption
Unit: mL
Control
Liv-*Has2*
D
V_{O2}
V_{CO2}
Unit: ml/h/kg
Control
Liv-*Has2*
E
Weight gain on Dox50 HFHS
Unit: gram
**
Ctrl
Liv-*Has2*
F
dHDL
Unit: mg/dL
Cholesterol
Unit: mg/dL
Triglyceride
Unit: mg/dL
NEFA
Unit: mEq/L
Ctrl
Liv-*Has2*
G
Glucose Tolerance Test
Control
Liv-*Has2*
Glucose (mg/dL)
0 min
15 min
30 min
60 min
120 min
H
AST
Unit: U/L
ALT
Unit: U/L
Ctrl
Liv-*Has2*

Supplementary Fig. 5: Hepatic *Has2* overexpression on systemic metabolism

- A: Doxycycline dose-dependent increase of serum HA in Liv-Has2 mice after five days of doxycycline HFHS diet treatment. Each data point is plotted on the graph. (n = 13, 2, 2, 2, 2, 3, 1 mice for HFHS diet containing 0, 1, 2.5, 5, 10, 25, 50 mg/kg doxycycline, respectively).
- B: Body weight, fat mass and lean mass before and after the metabolic cage experiment referred in Fig 6D (n = 12 mice per genotype).
- C: Accumulative food intake and water consumption during the metabolic cage experiment referred in Fig 6D (n = 12 mice per genotype).
- D: O_2 consumption (VO_2) and CO_2 production (VCO_2) during the metabolic cage experiment referred in Fig 6D (n = 12 mice per genotype).
- E: Weight gain for Liv-Has2 mice treated with Dox50 HFHS diet for four weeks (n = 9 mice per genotype). Two-tailed *t*-test, $p = 0.0013$.
- F: Serum lipids levels in Liv-*Has2* mice treated with Dox50 HFHS diet for four weeks (n = 4 mice for control, n = 3 mice for Liv-*Has2*).
- G: Glucose tolerance for Liv-*Has2* mice treated with Dox50 HFHS diet for four weeks. (n = 4 mice for control, n = 3 mice for Liv-*Has2*).
- H: Serum AST and ALT activities in Liv-*Has2* mice treated with Dox50 HFHS diet for four weeks (n = 4 mice for control, n = 3 mice for Liv-*Has2*).

All data are presented as Mean ± s.e.m. ** indicates $p \leq 0.01$. Two-tailed *t*-test (F) (H). Two-way ANOVA (B) (C) (D) (G).

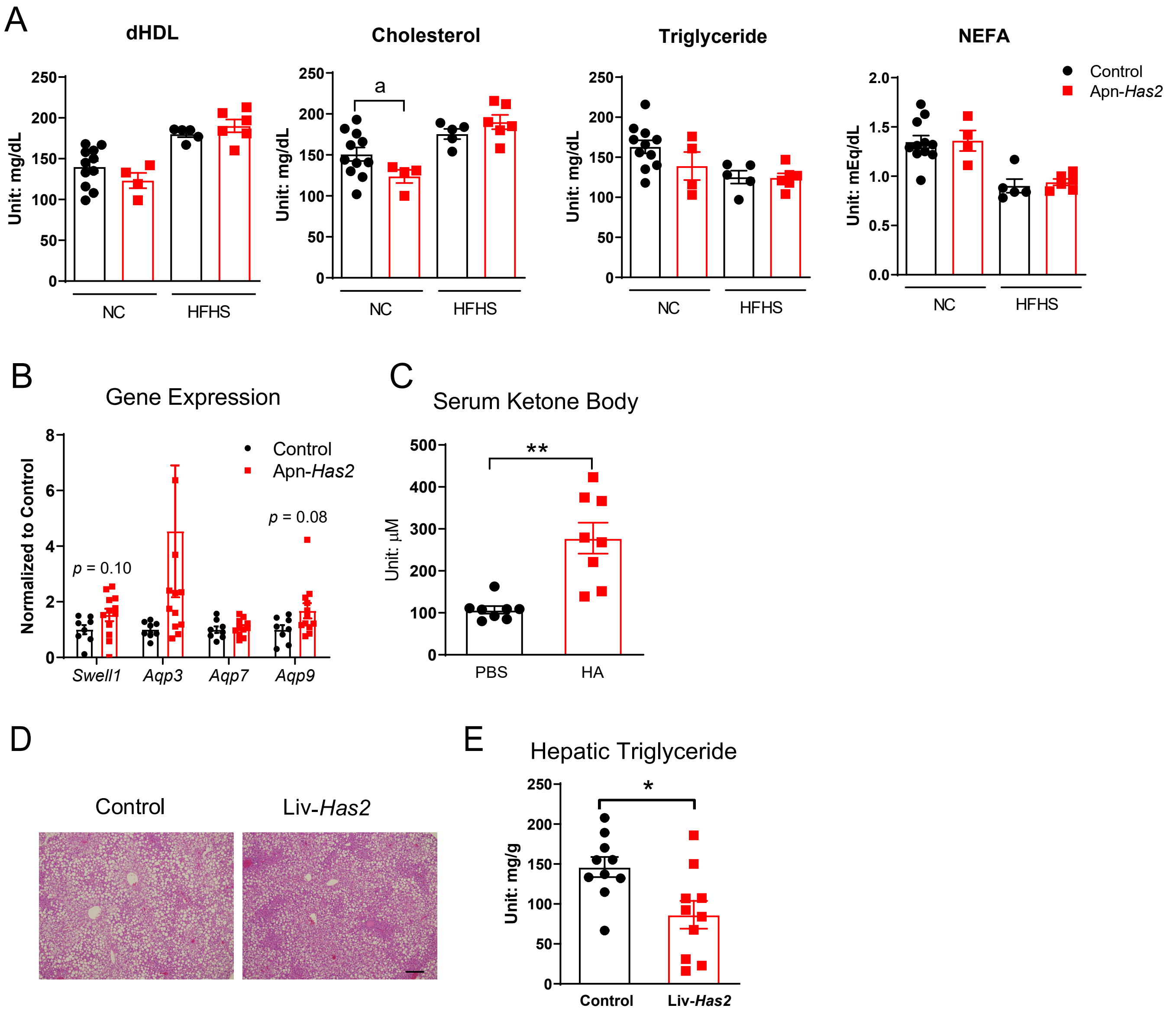

A
dHDL
Cholesterol
Triglyceride
NEFA
Control
Apn-Has2
Unit: mg/dL
Unit: mEq/dL
a
NC
HFHS
B
Gene Expression
Normalized to Control
p = 0.10
p = 0.08
Swell1
Aqp3
Aqp7
Aqp9
C
Serum Ketone Body
Unit: µM
**
PBS
HA
D
Control
Liv-Has2
E
Hepatic Triglyceride
Unit: mg/g
*
Control
Liv-Has2

Supplementary Fig. 6: Additional metabolic characterization of Apn-*Has2* and Liv-*Has2* mice

A: dHDL, total Cholesterol, triglyceride and total NEFA levels in Apn-*Has2* mice on Dox600 chow diet and Dox600 HFHS diet for 12 weeks. (n = 11 mice for control on normal diet, n = 4 mice for Apn-*Has2* on normal diet, n = 5 mice for control on HFHS diet, n = 6 mice for Apn-*Has2* on HFHs diet). 2way ANOVA followed by Sidak's multiple comparisons test. a = 0.1058.

B: Gene expression in adipose tissue overexpressing *Has2* after 5 days of Dox600 chow diet treatment (n = 8 mice for control, n = 12 mice for Apn-*Has2* mice). One sample in Apn-Has2 group was not detected for *Aqp7* gene. Multiple two-tailed *t*-test.

C: Serum total ketone body concentration after 4-week HA treatment outlined in Fig. 5E (n = 8 mice per treatment). Two-tailed *t*-test, $p = 0.0005$.

D: Representative liver histology of Liv-*Has2* mice after 16 weeks Dox10 HFHS treatment. Scale bar = 100 μm.

E: Hepatic triglyceride contents of Liv-*Has2* mice after 16 weeks Dox10 HFHS treatment (n = 10 per genotype). Two-tailed *t*-test, $p = 0.0121$.

All data are presented as Mean ± s.e.m. * indicates $p \leqslant 0.05$, ** indicates $p \leqslant 0.01$.

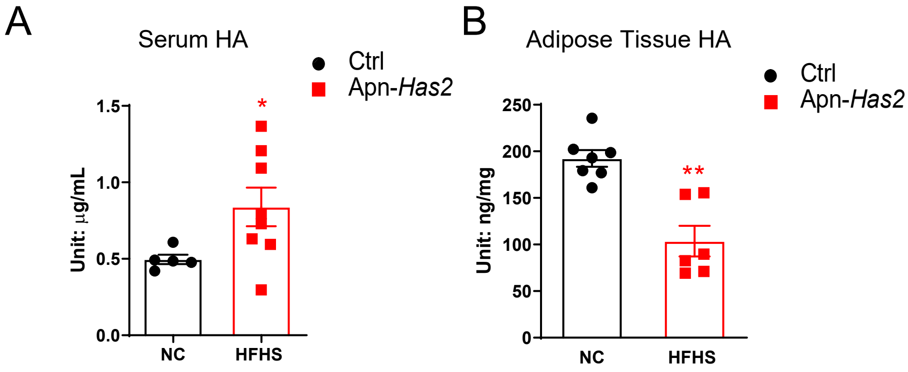
A
Serum HA
Ctrl
Apn-*Has2*
Unit: μg/mL
1.5
1.0
0.5
0.0
*
NC
HFHS
B
Adipose Tissue HA
Ctrl
Apn-*Has2*
Unit: ng/mg
250
200
150
100
50
0
**
NC
HFHS

Supplementary Fig. 7: Chronic HFHS treatment on serum HA and adipose tissue HA levels

A: Serum HA levels after 16 weeks of dietary treatment. (n = 5 mice for normal chow, n = 8 mice for HFHS diet). Two-tailed *t*-test, $p = 0.0311$.

B: Inguinal adipose tissue HA levers after 16 weeks of dietary treatment. (n=7 for normal chow, n=6 for HFH diet). Two-tailed *t*-test, $p = 0.0004$.

All data are presented as Mean ± s.e.m. * indicates p $\leqslant$ 0.05, ** indicates p $\leqslant$ 0.01.

Supplementary Table 1: List of primers used in the paper.

Name of the Gene	Name of the primer	Sequence (5' to 3')
Has1	Has1-f	GGCGAGCACTCACGATCATC
	Has1-r	AGGAGTCCATAGCGATCTGAAG
Has2	Has2-f	TGTGAGAGGTTTCTATGTGTCCT
	Has2-r	ACCGTACAGTCCAAATGAGAAGT
Has3	Has3-f	CTAGCCTTCCTAGTCTCTGG
	Has3-r	GGCTATACTGTTCTGGCTTC
Col1a1	Col1a1-f	GATGGATTCCCGTTCGAGTA
	Col1a1-r	ATGTAGGCTACGCTGTTCTT
Col1a2	Col1a2-f	TACAACGTAGAAGGGGTGTC
	Col1a2-r	GTGATGTTCTGAGAAGCACG
Col4a1	Col4a1-f	TGTGGATCGGCTATTCCTTC
	Col4a1-r	GCTTCTTGAACATCTCGCTT
Col4a3	Col4a3-f	CACTGGTACAAGAATGCGAG
	Col4a3-r	ATGTGCACGTTTGTTTCCTT
Col4a5	Col4a5-f	TCAAACAACAGAAGCACCAC
	Col4a5-r	GAAAGGCATGGTACTGAAGC
Aqp7	Aqp7-f	GTTTTGGATTCGGAGTGACC
	Aqp7-r	CCCAGCACATATACAGGGAA
Ucp1	UCP1-f	ACTGCCACACCTCCAGTCATT
	UCP1-r	CTTTGCCTCACTCAGGATTGG
Adrb1	Adrb1-f	GAACCCTGCAACCTGTCGTC
	Adrb1-r	CCAGCAGTAGGCCCATACC
Adrb2	Adrb2-f	GGGAACGACAGCGACTTCTT
	Adrb2-r	GCCAGGACGATAACCGACAT
Adrb3	Adrb3-f	CCTTGGGCGAAACTGGTTG
	Adrb3-r	GTTGGTGACAGCTAGGTAGCG
Pde3b	Pde3b-f	AAAGCGCAGCCGGTTACTAT
	Pde3b-r	CACCACTGCTTCAAGTCCCAG
Pnpla2	Atgl-f	CAACGCCACTCACATCTACGG

	Atgl-r	GGACACCTCAATAATGTTGGCAC
Lipe	Hsl-f	CCAGCCTGAGGGCTTACTG
	Hsl-r	CTCCATTGACTGTGACATCTCG
Ckmt1	Ckmt1-f	TGAGGAGACCTATGAGGTATTTGC
	Ckmt1-r	TCATCAAAGTAGCCAGAACGGA
Ckmt2	Ckmt2-f	GCATGGTGGCTGGTGATGAG
	Ckmt2-r	AAACTGCCCGTGAGTAATCTTG
Atp2a2	Atp2a2-f	GAGAACGCTCACACAAAGACC
	Atp2a2-r	CAATTCGTTGGAGCCCCAT
Pm20d1	Pm20d-f	CTTCTCTTTTTCGCTACGGTCT
	Pm20d-r	CACCTTTCAGCGCCTCTTTTAT
Gdf15	Gdf15-f	CTGGCAATGCCTGAACAGCG
	Gdf15-r	GGTCAGGACTTGGTTCTGAG
Fgf21	Fgf21-f	CTGCTGGGGGTCTACCAAG
	Fgf21-r	CTGCGCCTACCACTGTTCC
Ppargc1a	PGC1a-f	AGCCGTGACCACTGACAACGAG
	PGC1a-r	GCTGCATGGTTCTGAGTGCTAAG
Adipoq	Apn-f	TGTTCCTCTTAATCCTGCCCA
	Apn-r	CCAACCTGCACAAGTTCCCTT
Fabp4	Fabp4-f	AAGGTGAAGAGCATCATAACCCT
	Fabp4-r	TCACGCCTTTCATAACACATTCC
Cebpa	Cebpa-f	CAAGAACAGCAACGAGTACCG
	Cebpa-r	GTCACTGGTCAACTCCAGCAC
Pparg	Pparg-f	TCGCTGATGCACTGCCTATG
	Pparg-r	GAGAGGTCCACAGAGCTGATT
