# Supplementary material for: Adipose tissue hyaluronan production improves systemic glucose homeostasis and primes adipocytes for CL 316,243-stimulated lipolysis
Source: Nat Commun. 2021 Aug 10;12:4829. doi: 10.1038/s41467-021-25025-4 (PMC8355239; doi:10.1038/s41467-021-25025-4)
Supplement: Supplementary file 2 — Reporting Summary [file 41467_2021_25025_MOESM2_ESM.pdf]

nature research

Corresponding author(s): Philipp E. Scherer

Last updated by author(s): Mar 12, 2021

Reporting Summary

Nature Research wishes to improve the reproducibility of the work that we publish. This form provides structure for consistency and transparency in reporting. For further information on Nature Research policies, see our Editorial Policies and the Editorial Policy Checklist.

Statistics

For all statistical analyses, confirm that the following items are present in the figure legend, table legend, main text, or Methods section.

n/a	Confirmed	
☐	☒	The exact sample size (*n*) for each experimental group/condition, given as a discrete number and unit of measurement
☐	☒	A statement on whether measurements were taken from distinct samples or whether the same sample was measured repeatedly
☐	☒	The statistical test(s) used AND whether they are one- or two-sided<br>*Only common tests should be described solely by name; describe more complex techniques in the Methods section.*
☐	☒	A description of all covariates tested
☐	☒	A description of any assumptions or corrections, such as tests of normality and adjustment for multiple comparisons
☐	☒	A full description of the statistical parameters including central tendency (e.g. means) or other basic estimates (e.g. regression coefficient) AND variation (e.g. standard deviation) or associated estimates of uncertainty (e.g. confidence intervals)
☐	☒	For null hypothesis testing, the test statistic (e.g. *F*, *t*, *r*) with confidence intervals, effect sizes, degrees of freedom and *P* value noted<br>*Give P values as exact values whenever suitable.*
☒	☐	For Bayesian analysis, information on the choice of priors and Markov chain Monte Carlo settings
☒	☐	For hierarchical and complex designs, identification of the appropriate level for tests and full reporting of outcomes
☒	☐	Estimates of effect sizes (e.g. Cohen's *d*, Pearson's *r*), indicating how they were calculated

Our web collection on statistics for biologists contains articles on many of the points above.

Software and code

Policy information about availability of computer code

Data collection	Odyssey Version 3.0
Data analysis	Odyssey Version 3.0, FlowJo Version 10.2

For manuscripts utilizing custom algorithms or software that are central to the research but not yet described in published literature, software must be made available to editors and reviewers. We strongly encourage code deposition in a community repository (e.g. GitHub). See the Nature Research guidelines for submitting code & software for further information.

Data

Policy information about availability of data

All manuscripts must include a data availability statement. This statement should provide the following information, where applicable:

- Accession codes, unique identifiers, or web links for publicly available datasets
- A list of figures that have associated raw data
- A description of any restrictions on data availability

All data supporting the findings of this study are available within the paper. Mouse models are available from the corresponding author on request.

Field-specific reporting

Please select the one below that is the best fit for your research. If you are not sure, read the appropriate sections before making your selection.

☒ Life sciences ☐ Behavioural & social sciences ☐ Ecological, evolutionary & environmental sciences

For a reference copy of the document with all sections, see nature.com/documents/nr-reporting-summary-flat.pdf

Life sciences study design

All studies must disclose on these points even when the disclosure is negative.

Sample size	No statistical methods were used to determine sample size. Sample size was determined empirically or from sizes commonly published for the specific type of experiment. For all mouse studies, the n value corresponds to individual mice of a given treatment. For cell culture experiments, the n values correspond to the number of replicates in the experiment.
Data exclusions	Only data points generated in technically failed experiments were excluded. No data points were excluded using statistical methods.
Replication	Studies employing mice were not replicated, unless shown within this manuscript, due to ethical, logistical and financial limitations. All in vitro studies were repeated at least twice with similar results, except 2-DG glucose uptake ,due to limited reagents and environmental concerns of using radioactive materials.
Randomization	All studies were performed with randomization when necessary. All animal groups were randomly assigned prior to treatment or experimentation. For in vitro studies, cultures were randomly assigned experimental conditions.
Blinding	All data analysis was conducted in a blinded manner. All in vivo experiments were conducted in a blinded manner. In vitro experiments were not blinded due to logistical issues.

Reporting for specific materials, systems and methods

We require information from authors about some types of materials, experimental systems and methods used in many studies. Here, indicate whether each material, system or method listed is relevant to your study. If you are not sure if a list item applies to your research, read the appropriate section before selecting a response.

Materials & experimental systems

n/a	Involved in the study	
☐	☒	Antibodies
☐	☒	Eukaryotic cell lines
☒	☐	Palaeontology and archaeology
☐	☒	Animals and other organisms
☐	☒	Human research participants
☒	☐	Clinical data
☒	☐	Dual use research of concern

Methods

n/a	Involved in the study	
☒	☐	ChIP-seq
☒	☐	Flow cytometry
☒	☐	MRI-based neuroimaging

Antibodies

Antibodies used

Antibody Source Catalog number Dilution Application
HAS2, Santa Cruz Biotechnology, sc-34068 1:200 WB
HSL, Santa Cruz Biotechnology, sc-74489 1:200 WB
α-Tubulin, Santa Cruz Biotechnology, sc-53030 1:200 WB
Actin, Sigma, #A4700 1:1000 WB
Adiponectin, homemade 1:1000 WB
Goat anti-Mouse IRDye 680RD Li-cor 926-68070 1:10,000 WB
Goat anti-rabbit IRDye 800CW Li-cor 925-32211 1:10,000 WB
Goat anti-Rat DyLight 800 Thermo Fisher SA5-10024 1:10,000 WB

Validation

Antibodies were validated by the information provided by the vendor (see below). Adiponectin antibody was validated through western blot of adiponectin whole body knockout mouse serum.
HAS2, Santa Cruz Biotechnology, sc-34068 https://www.scbt.com/p/has2-antibody-y-14
HSL, Santa Cruz Biotechnology, sc-74489 https://www.scbt.com/p/hsl-antibody-g-7
α-Tubulin, Santa Cruz Biotechnology, sc-53030 https://www.scbt.com/p/alpha-tubulin-antibody-yol1-34
Actin, Sigma, #A4700 https://www.sigmaaldrich.com/catalog/product/sigma/a4700?lang=en&region=US

Eukaryotic cell lines

Policy information about cell lines

Cell line source(s)	Met-1(fvb2) (RRID:CVCL_U373), acquired from Lonza (#1227), HEK-293 (ATCC® CRL-1573™), acquired from ATCC
Authentication	None of the cell line used were authenticated
Mycoplasma contamination	The cell line was not tested for mycoplasma contamination
Commonly misidentified lines (See ICLAC register)	No commonly misidentified cell lines were used in this study.

Animals and other organisms

Policy information about studies involving animals; ARRIVE guidelines recommended for reporting animal research

Laboratory animals	Experiments were conducted with mice age between 6-34 weeks, dependent on the duration of study. All mice used for these studies were on the pure C58BL/6J background and housed in a specific pathogen free facility under standard conditions of temperature (~22-degree Celsius) with 12-hour light/dark cycle, food and water available ad lib. Cages and water were changed every week. Adiponectin-rtTA, TRE-Has2 mice were generated in the lab. These mice were crossed to R26-M2rtTA (The Jackson Laboratory, B6.Cg-Gt(ROSA)26Sortm1(rtTA*M2)Jae/J, Strain: 006965), liver-specific albumin-Cre transgenic (The Jackson Laboratory, B6.Cg-Speer6-ps1Tg(Alb-cre)21Mgn/J, Stock No: 003574) or Rosa26-loxP-STOP-loxP-rtTA transgenic (The Jackson Laboratory, Gt(ROSA)26Sortm1(rtTA,EGFP)Nagy/J, Stock No: 005572) mice, as described in the manuscript.
Wild animals	No wild animals were used in this paper
Field-collected samples	No field-collected samples were used in this paper
Ethics oversight	Institutional Animal Care and Use Committee of the University of Texas Southwestern Medical Center (Protocol number 2015-101207G) and Baylor College of Medicine (Protocol number AN-8158).

Note that full information on the approval of the study protocol must also be provided in the manuscript.

Human research participants

Policy information about studies involving human research participants

Population characteristics	Subject characteristics are summarized in Table 1.
Recruitment	Participants were recruited using the Volunteers for Health database at Washington University School of Medicine and by local postings between April 2016 and September 2017.
Ethics oversight	This study was approved by the Human Research Protection Office of Washington University (201512086)

Note that full information on the approval of the study protocol must also be provided in the manuscript.
